# Target prediction utilising negative bioactivity data covering large chemical space

**DOI:** 10.1186/s13321-015-0098-y

**Published:** 2015-10-24

**Authors:** Lewis H. Mervin, Avid M. Afzal, Georgios Drakakis, Richard Lewis, Ola Engkvist, Andreas Bender

**Affiliations:** Department of Chemistry, Centre for Molecular Informatics, University of Cambridge, Lensfield Road, Cambridge, CB2 1EW UK; Discovery Sciences, Chemistry Innovation Centre, AstraZeneca R&D, 43183 Mölndal, Sweden

**Keywords:** Cheminformatics, QSAR, Similarity search, Data mining, BEDROC

## Abstract

**Background:**

In silico analyses are increasingly being used to support mode-of-action investigations; however many such approaches do not utilise the large amounts of inactive data held in chemogenomic repositories. The objective of this work is concerned with the integration of such bioactivity data in the target prediction of orphan compounds to produce the probability of activity and inactivity for a range of targets. To this end, a novel human bioactivity data set was constructed through the assimilation of over 195 million bioactivity data points deposited in the ChEMBL and PubChem repositories, and the subsequent application of a sphere-exclusion selection algorithm to oversample presumed inactive compounds.

**Results:**

A Bernoulli Naïve Bayes algorithm was trained using the data and evaluated using fivefold cross-validation, achieving a mean recall and precision of 67.7 and 63.8 % for active compounds and 99.6 and 99.7 % for inactive compounds, respectively. We show the performances of the models are considerably influenced by the underlying intraclass training similarity, the size of a given class of compounds, and the degree of additional oversampling. The method was also validated using compounds extracted from WOMBAT producing average precision-recall AUC and BEDROC scores of 0.56 and 0.85, respectively. Inactive data points used for this test are based on presumed inactivity, producing an approximated indication of the true extrapolative ability of the models. A distance-based applicability domain analysis was also conducted; indicating an average Tanimoto Coefficient distance of 0.3 or greater between a test and training set can be used to give a global measure of confidence in model predictions. A final comparison to a method trained solely on active data from ChEMBL performed with precision-recall AUC and BEDROC scores of 0.45 and 0.76.

**Conclusions:**

The inclusion of inactive data for model training produces models with superior AUC and improved early recognition capabilities, although the results from internal and external validation of the models show differing performance between the breadth of models. The realised target prediction protocol is available at https://github.com/lhm30/PIDGIN.

**Graphical abstract:**
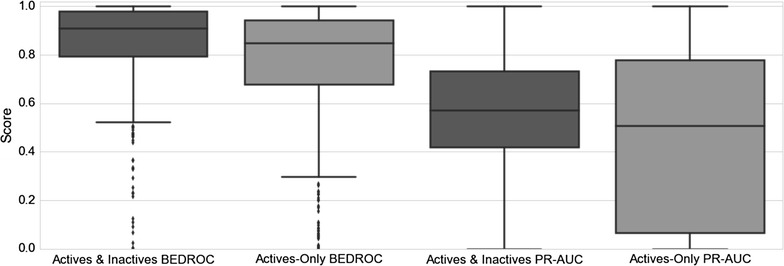
The inclusion of large scale negative training data for in silico target prediction improves the precision and recall AUC and BEDROC scores for target models.

**Electronic supplementary material:**

The online version of this article (doi:10.1186/s13321-015-0098-y) contains supplementary material, which is available to authorized users.

## Background

A principle challenge faced by the information gleaned from phenotypic screening is that many of the assessed compounds remain orphan ligands, as the respective mode-of-action (MOA) remains undetermined in the first instance [1]. Consequently, the subsequent identification of the modulated targets for active compounds, known as ‘target deconvolution’, is required [2]. Various biochemical affinity purification methods provide direct approaches to discover target proteins binding small molecules of interest for this purpose [3–6]. Although these elucidation experiments can explicitly determine compound-target interactions, such procedures are costly and time-consuming [4, 7, 8]. These procedures require large amounts of protein extract and stringent experimental conditions, while many techniques have also been deemed best suited for situations where a high affinity ligand binds to a protein [1, 9]. Other difficulties involve the challenge of preparing and immobilizing affinity reagents (targets) that still retain their cellular activity (i.e. ensuring that proteins still interact with the small molecule while it is bound to the solid surface) [10]. Such caveats are responsible for the increased interest in novel computational target deconvolution strategies for drug discovery [11].

In silico protein target prediction is a well-established computational technique that offers an alternative avenue to infer target-ligand interactions by utilizing known bioactivity information [12]. These methods have played an important role in the field of efficacy prediction and the prediction of toxicity [13–17]. Such approaches are designed to predict targets for orphan compounds early in the drug development phase, with the predictions forming the base of an experimental confirmation afterwards. Both structure-based and ligand-based methods exist for the prediction of protein targets for small molecule ligands [12, 18–22]. The former methods generally describe approaches that exploit the structural information of the protein, combined with scoring functions, in an attempt to predict ligand–target pairs [23–25]. Ligand-based methods investigate an identified structure–activity relationship (SAR) space, using similarity searching on large numbers of annotated protein–ligand pairs obtained from chemogenomic databases [26–29]. Such predictive models are founded upon the principle of chemical similarity, relying on the relevant similarities of compound features from targets which are likely to be responsible for binding activity [30, 31]. The focus of this work is concerned with improving current ligand-based methods for target prediction.

Similarity searching for ligand-based target prediction is considered the simplest form of in silico target prediction and has long been established within the literature [12, 17, 22, 27, 32–38]. In these models, predictions are based on the principle of molecular similarity to identified bioactive compounds from chemogenomic databases [31, 35]. The simplistic nature of these methods means that they are only able to consider the structure of a molecule as a whole, which significantly hinders the predictive power of these models. Building on this theme, data-mining algorithms are capable of considering multiple combinations of compound fragments by applying pattern recognition techniques. They have gained traction in target prediction due to their demonstrated ability to more efficiently extrapolate predictions [12], and are less time consuming when compared to other approaches such as molecular docking [20]. One of the earliest and most widely used examples of data-mining target elucidation is the continuously curated and expanded Prediction of Activity Spectra for Substances (PASS) software [21], which was assimilated from the bioactivites of more than 270,000 compound-ligand pairs. The authors trained the model on multilevel neighbourhoods of atoms (MNA) descriptors, producing predictions based on Bayesian estimates of probabilities.

The Naïve Bayes (NB) classifiers are a popular family of algorithms used for the prediction of bioactivity for compounds [39–42]. These methods offer a quick training and prediction times and are relatively insensitive to noise [43]. Nidhi et al. [40], employed a multi-class Naïve Bayes classification algorithm trained on a data set comprised of over 960 target proteins extracted from the ‘World Of Molecular BioAcTivity’ (WOMBAT) database [44]. Another target prediction algorithm developed by Koutsoukas et al. [39], is able to predict structure activity relationships (SARs) for orphan compounds using either a Laplacian-modified Naïve Bayes classifier or a Parzen-Rosenblatt Window (PRW) learner. The algorithm was trained on more than 155,000 ligand–protein pairs from the ChEMBL14 database [45], encompassing 894 different human protein targets. After benchmarking experiments [39], it was found that the PRW learner outperformed the Naïve Bayes algorithm overall, achieving a recall and precision of 66.6 and 63.3 %, respectively.

Support Vector Machines (SVMs) have also been employed for task of target prediction. These models have utilised smaller amounts of data in comparison to NB models, mostly due to the computational expense of the SVM algorithm. Examples of SVM methods include a model obtaining more than 80 % sensitivity consistently for five different protein targets [42]; however the limitation of this implementation to five molecular targets only allows for a very narrow view of compound activity. Nigsch et al. [28]. investigated the use of a Winnow algorithm for target prediction and compared the learner to the performance of a Naïve Bayes model, comprising a set of 20 targets extracted from WOMBAT. The comparison of the overall performance of the two algorithms did not yield significant differences, but the overall findings supported the view that ensembled machine-learning models could be used to yield superior predictions.

Ligand-based predictions provide a basis for further analysis when attempting to rationalise mechanism-of-action. Liggi et al. [46], extended the predictions from a PRW target prediction algorithm, by annotating enriched targets implicated with a cytotoxic phenotype, using associated pathways from the GO [47, 48] and KEGG [49, 50] pathway databases. The annotation of enriched targets with pathways gave improved insight into understanding the MOA of the phenotype, underlining the signalling pathways involved in cytotoxicity. Other examples include the combination of target prediction and classification tree techniques for rationalising phenotypic readouts from a rat model for hypnotics [51]. The authors derived interpretable decision trees for the observed phenotypes and inferred the combination of modulated targets which contribute towards good sleeping patterns. A review of the known mechanisms of hypnotics suggested that the results were consistent with current literature in many cases.

Such publications illustrate the real-world application of predictions obtained from in silico target prediction methods for both on-target and off-target bioactivity predictions [14]. Although most of these methods produce a probability of activity for an orphan compound against a given target, the approaches mentioned here do not utilise inactive bioactivity data [39]. The objective of this work is hence concerned with the construction of an in silico target prediction approach that is able to consider both the probability for activity and inactivity of orphan compounds against a range of biological targets, thus, giving a more holistic perspective of chemical space for factors that contribute and counteract bioactivity.

The realized target prediction tool is validated both through cross-validation and an external validation set. Five-fold cross validation was employed to evaluate the model and was also used to generate class-specific activity thresholds, based on the optimum cut-off value for a range of different metrics. The performance of the tool when applied to test data extracted from the WOMBAT database is also evaluated. This provides insight into the models ability to extrapolate predictions to a real-world setting. Finally, a comparison to a model generated solely from activity data was also conducted to evaluate the value of the incorporation of inactivity data in the models.

## Results and discussion

### Internal validation results

**Fig. 1:**
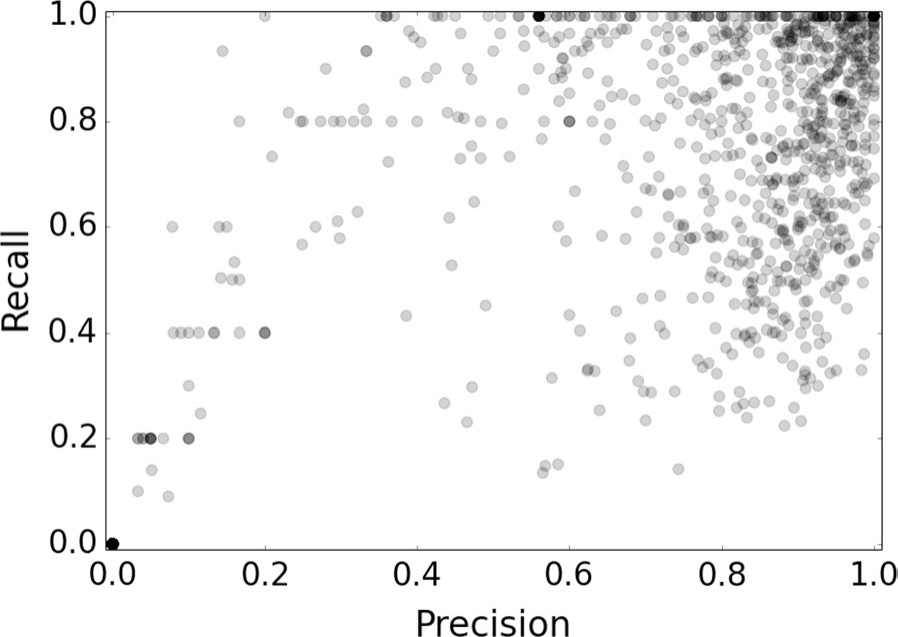
Distribution of Precision/Recall values achieved by the models during fivefold cross-validation. Many of the models populate high Precision and Recall values towards the *top right* of the plot

Precision and recall values were computed for each of the fivefolds and averaged to give a single metric to measure the performance each of the 1080 target classification classes (Fig. 1). Overall, the models achieve a mean recall and precision of 67.7 and 63.8 % for actives, and 99.6 and 99.7 % for inactive compounds. Internal validation exhibits differing performance profiles across the breadth of models. 172 of the targets failed to predict any active molecules and so received precision and active recall values of 0, with 150 of these (87.2 %) comprising target classification classes comprising fewer than 20 active training compounds. We also considered a fivefold cross validation splits a set of 20 compounds into 5 subsets of 4 compounds each, so it is unsurprising that the models do not maintain their predictive ability for small classes.

**Fig. 2:**
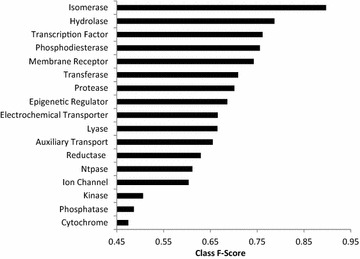
Performance of different target classes. The *top* performing classes tend to contain low numbers of targets i.e. The *top* 3 ranked classes, isomerase, ligase and aminoacyltransferase all comprise fewer than 5 targets

Figure 2 depicts how the performance of the models differs by target class. The 3 highest performing targets tend to be smaller groups of enzymes comprising fewer than 5 proteins, for example Ligases, Isomerases and Aminoacyltransferase all achieve average F_1_-Score performances above 0.8. The transcription factor class is the highest performing set of targets with a count above 30, averaging a F_1_-Score of 0.76. In comparison, Kinases comprise one of the largest classification classes of targets (273), which have comparatively low performance of 0.50. This target class has been previously shown to be problematical for in silico target prediction due to the promiscuity of the targets [39]. In these cases, these models have difficulty with predictions due to increased dissimilarity within the active training sets and increased similarity between active and inactive training sets, meaning that the models can not identify signals responsible for activity.

The high density of points towards the top right of the plot of Fig. 1 depicts that a significant number of models obtained high precision and recall values. Upon further investigation, it was found that 141 of the 157 targets (89.8 %) that score precision and recall scores above 0.97 belong to targets comprised of sphere excluded (SE) inactive compounds. Such a high performance is a result of the sphere exclusion (SE) algorithm that requires that molecules must be suitably dissimilar from actives. In these cases, the SE inactive compounds can more easily be distinguished apart actives in comparison to PubChem inactive compounds during cross validation. Conversely, due to the absence of a dissimilarity selection requirement, experimentally confirmed inactive compounds from PubChem are likely to be more skeletally similar to actives from ChEMBL, as inactive compounds tend to originate from structurally similar scaffolds to actives (Additional file 1: Table S1). This trend blurs the boundary of the hyperplane between the active and inactive classes. The results from internal validation also indicate that the models frequently perform with low recall, which is most likely a consequence of the class imbalance of the data, when active compounds are predicted as false negatives due to the apparent over representation of features in the inactive classes.

**Fig. 3:**
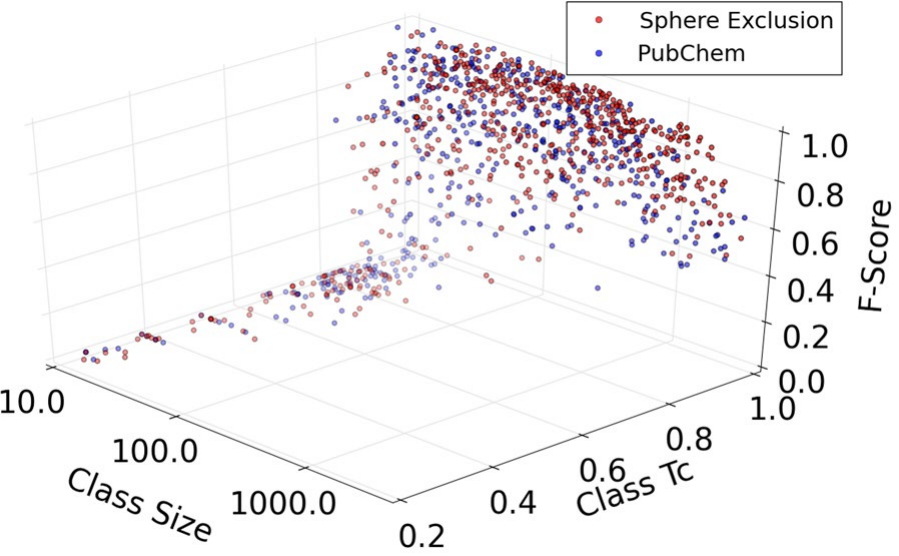
Effect of number of active compounds (Class size) and intra-target Tanimoto similarity (Class Tc) on F_1_ Score performance. Sphere excluded targets perform better in comparison to under-sampled targets. Targets with high Tc and consisting of more than 40 active compounds perform well overall. In contrast, targets comprising a small number of active compounds, e.g. 40 or fewer, display a large variation in target similarity and performance. Marker colour intensity represents depth in 3D space (transparency indicates markers that are further away). Class Tc is calculated using the average of the top 5 nearest neighbours of the training compounds

The influence of class size, Top 5 nearest intraclass neighbours and F-score performance are explored in Fig. 3. Here it is demonstrated that both increasing class similarity and size are shown to improve the predictions for the models, as the majority of classes larger than 40 compounds are dominated by high Tc values between 0.9 and 1.0 (data points past this point tend to line the back wall of the plot). In comparison, smaller models below 40 compounds are shown to have higher variance in the nearest neighbour similarity due to the smaller coverage of chemical space (bottom left), with Tc values ranging between 0.2 and 0.99. The intraclass similarity of the models increases in accordance with target size as the likelihood of including a similar neighbour increases with a greater coverage of chemical space. In the case of small model sizes, models with low Tc values perform poorly (as the chance of retaining similar compounds throughout the folds decreases), while small target models comprising similar nearest neighbours perform better. This evidence supports the view that smaller sized classes below 40 compounds can be reliably utilized, providing that similar compounds are represented within the training sets. The figure also shows the influence of the SE algorithm on the performance of the models, with the red and blue markers denoting PubChem-only inactive compounds and SE inactive compounds respectively. Targets with SE inactive compounds dominate among highly performing targets, with 73 of the 420 SE classes achieving precision values over 0.95. This can be seen via the density of red markers towards limit of the y axis. Interestingly, the figure illustrates that PubChem and SE classes are both performing poorly in situations with low intraclass similarity, with markers populating the base of the plot. In these circumstances, the chemical space covered by the models is too small and diverse, meaning that the NB algorithm is not able to establish distinguishing features between activity classes.

### External validation results

#### Validation using WOMBAT

The performance of the models was analysed using an external data set extracted from WOMBAT. It is important to recognize that the bioactivity information included for the compounds-protein associations contained in WOMBAT do not contain experimentally confirmed inactive compounds, and that molecules without an annotation for a target are simply considered as inactive for that protein. Although this assumption may be correct in many cases, there are likely to be certain instances when a compound may actually be active. If these compounds were correctly annotated as active by the target prediction tool (a ‘*false* false positive’, and hence a ‘true positive’), this prediction would be penalised as a false positive due to the assumptions applied by the test data.

**Fig. 4:**
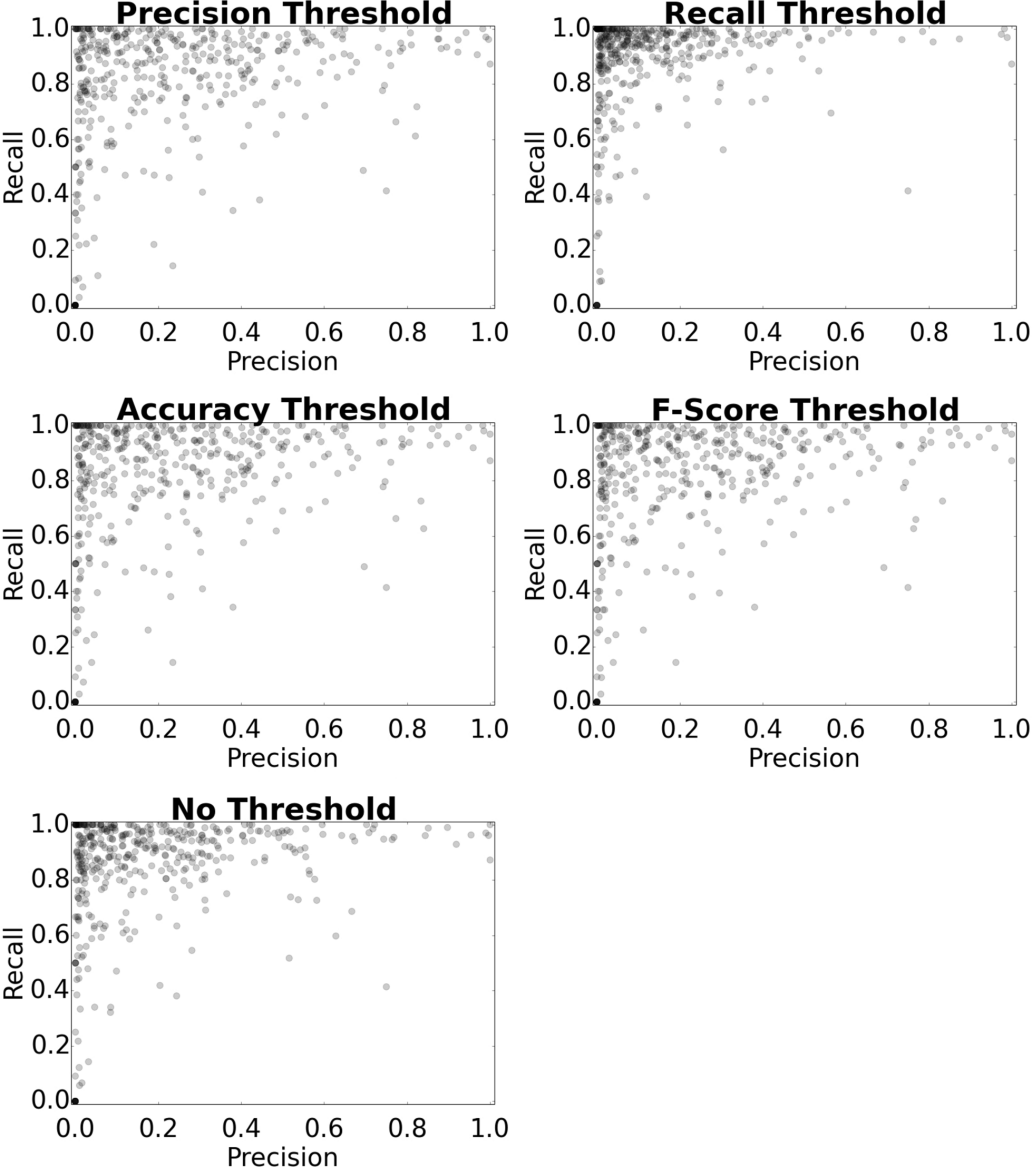
Precision and Recall distributions achieved by the models for the WOMBAT test data after application of thresholds. The precision, accuracy, F-score thresholds produce similar performance profiles, while the recall threshold produce a profile with elevated recall and lowered precision. Hence, different precision, accuracy and F-score metrics can be used to generate thresholds with high precision and lowered recall, while recall thresholds can be used to generate thresholds with improved recall at the cost of precision

Figure 4 and Table 1 illustrate the performance of the models upon application of the different thresholds to the predictions of the WOMBAT test data. The default threshold, which considers *Activity* when *P* (*activity*) > *P* (*inactivity*), produces an average precision and recall of 19.2 and 83.3 %. These findings exhibit a drop in performance that is experienced during external validation, for situations when orphan compounds are distinct from the training set. Compounds inhabiting dissimilar realms of chemical space are considered to be ‘near to’ or ‘outside’ the applicability domain, a key limitation of the models. The precision performance on the WOMBAT database is also reduced due to the failure of the some classes to predict any true-positive compounds, generating values of 0. The SE of negative samples in the training data is also likely to have limitations on its applicability on external test data, since compounds are sampled from a predefined area of therapeutically relevant chemical space.

**Table 1:** Average precision and recall for the different set of thresholds applied to WOMBAT

Threshold	Recall (%)	Precision (%)
Default 0.5	83.3	18.6
F1-Score	80.1	23.7
Accuracy	80.4	23.9
Precision	79.5	24.3
Recall	87.1	13.2

The Recall thresholds produce the most elevated recall with lowered precision, while the Precision threshold produces the highest precision whilst compromising recall

The precision values achieved by the models may initially seem low, but when this is compared to the values expected at random across all of the 1080 models (which would be in the area of ca. 1/1080, so about 0.1 % for each), the models are actually capable of retaining comparably high precision. The performance for the application of the F_1_-score, accuracy, precision and recall decision-based thresholds are also shown in Fig. 4. The results show a trade-off between precision and recall metrics for the models, with the F_1_-score, accuracy and precision metrics obtaining visually similar distributions. In comparison to the other metrics, the recall-based thresholds produce a distinctly separate profile with elevated recall and lowered precision, with many of the model performances populating an area of high recall and precision.

The varying performance profiles generated for each metric produce different model behaviour, and an ideal activity cut-off is dependent on the question proposed by a user and the application of the target predictions. For example, the stringent thresholds generated using the precision metric can be applied in cases such as candidate safety profiling, when outputs require few false negatives and accurate identification of dangerous compounds. Conversely, recall-based thresholds may be applied in circumstances requiring lenient assurance of predicted targets, perhaps during hit identification stages and mechanism-of-action studies. These thresholds will increase the chance of returning a more complete list of truly targeted proteins, at the cost of increasing the numbers of false positives.

**Fig. 5:**
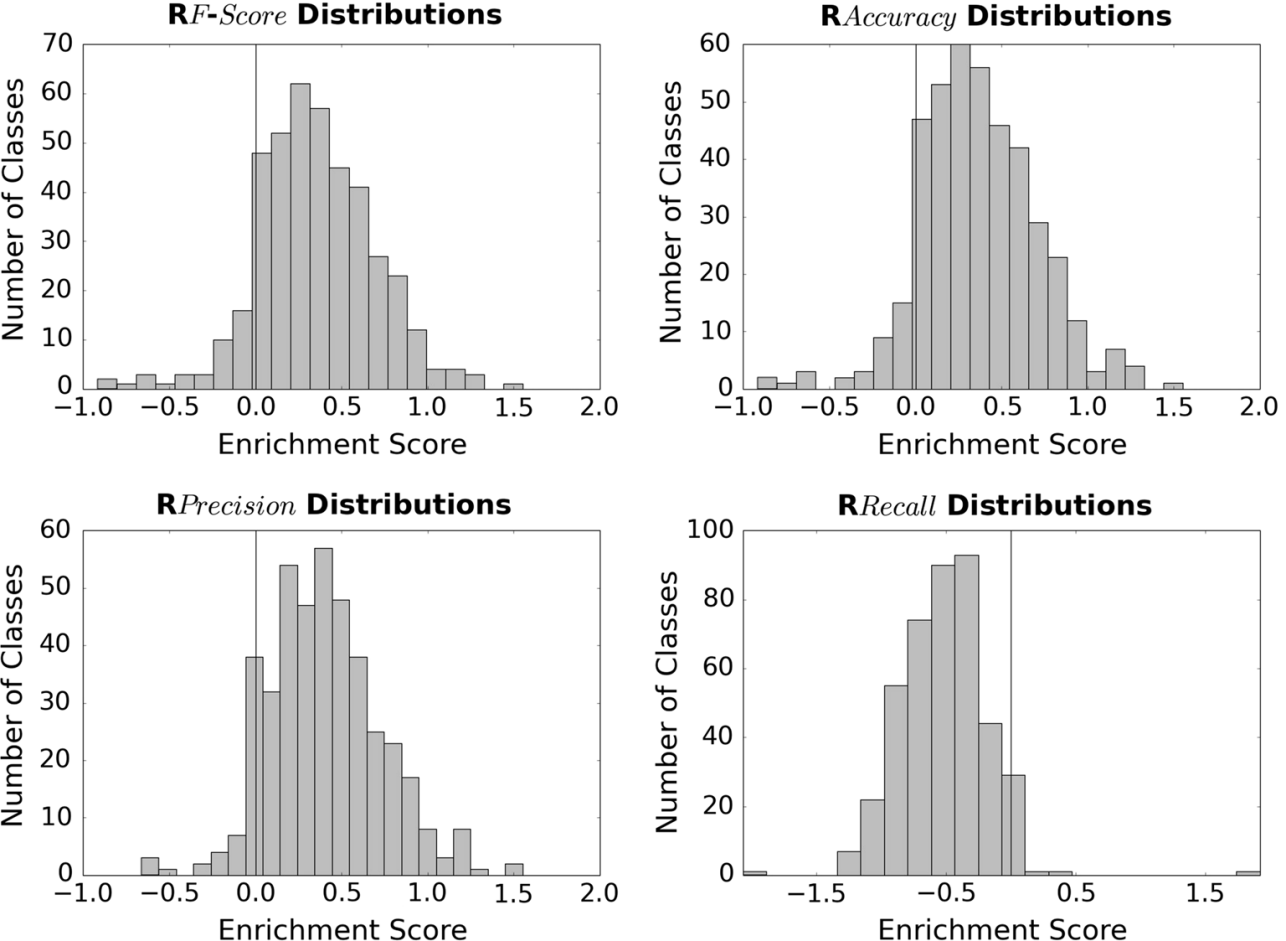
Distributions of R enrichment scores for the thresholds when applied to WOMBAT predictions. The F-score, accuracy and precision distributions show value in employing the thresholds when considering both recall and precision simultaneously, while the recall threshold produces a negative enrichment profile. Hence, the recall thresholds can be used when precision is not a predominant requirement

Table 2 and Fig. 5 display the results of the R score enrichment analysis, and demonstrate the similar performance profiles that are obtained using the F_1_, accuracy and precision decision thresholds. The positively skewed bell curves observed on these histogram plots peak around the same areas (approx. 0.3) with the majority of enrichment scores encompassing positive values, indicating that these thresholds frequently give improved F_1_-score performance. These findings mirror the previous conclusions that are drawn from Fig. 4 showing that the performance of these three sets of thresholds are similar. The enrichment score distributions for the recall-based thresholds glean a very different profile, exhibiting a negatively skewed bell curve with many negative enrichment values. These findings denote that many of the models achieved a higher F_1_ in the absence of this threshold, however this result does not indicate that this threshold metric is inferior to the other choices. Instead these results indicate that these values make for a poor choice when considering both recall and precision simultaneously (which is the case for the F_1_-score).

**Table 2:** A summary of decision threshold performance upon application to the WOMBAT database

Threshold	Average score	St. deviation	Median	Min	Max
F1 Score	0.349	0.341	0.333	−0.916	1.551
Accuracy	0.364	0.344	0.351	−0.916	1.551
Precision	0.402	0.327	0.380	−0.662	1.551
Recall	−0.527	0.330	−0.520	−2.064	1.918

F1 Score, accuracy and precision thresholds all glean similar performance values, while the recall shows a significantly separate profile with lowered performance values

The precision threshold is calculated to be the most enriched metric when considering the average and median enrichment scores for the models (Table 2). Upon application of this threshold boundary, 374 of the 418 targets benefitted, producing a positive enrichment score. Indeed, 154 of the targets show significantly high (≥0.5) enrichment values, revealing that the binary predictions produce considerably superior predictions in many cases. Overall, the enrichments in F_1_ values support the view that Precision-based thresholds provide the optimal metric when considering a medium between precision and recall.

### Analysis of the applicability domain

**Fig. 6:**
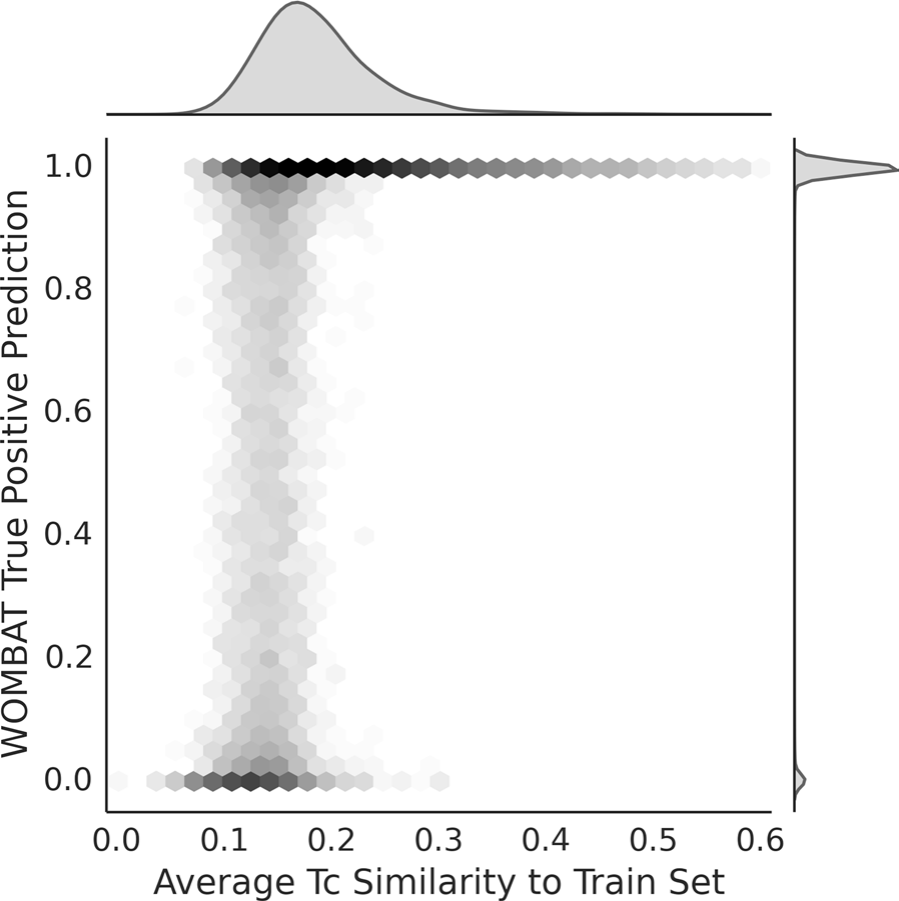
AD analysis of the True Positives from the WOMBAT test set. The models perform better overall when the similarity between the test and training sets are high. The area between 0.25 and 0.1 shows the largest variation in performance, when models can have problems distinguishing between activity classes. Similarities below 0.1 consistently predict true positives as inactive. Hence, a cut-off around 0.3 can be employed to give insight into the reliability of the predictions generated by models

This section aims to explore the AD through the relationship between the target prediction probabilities and the similarity of compounds of the training and WOMBAT test set. Figure 6 shows the relationship between the similarity among the test and training molecules and the consistency of the true-active target prediction probabilities. The figure illustrates that increasing the similarity between the training and test set appears to reduce the number of targets that are frequently underpredicted. A Tc over 0.3 tends to lead reliable predictions, with over 96 % of scores achieving 0.98 and above. In comparison, a region between 0.1 and 0.2 depicts a grey area, in which 59 thousand WOMBAT predictions obtain varied scores between 1 and 0 (a standard deviation of 0.34). Many of the scores span extreme probability values above 0.5 (50,758) and below 0.5 (9084) as shown by the density and histograms seen in the figure. This phenomenon is often expected as the absence or presence of a binary feature can heavily influence range of a prediction value when employing the Naïve Bayes algorithm [43]. The overall results from this plot suggest that a Tc cut-off of around 0.3 could be applied if a distance-based approach toward the AD is employed for this target prediction methodology. If a query molecule were above this threshold, this would indicate confidence in the reliability of the probabilities generated for the models. In comparison, a Tc value below this threshold would indicate that the probabilities produced by the models are not consistently dependable. Notably, the models are able to retain some good performance in conditions of low Tc values between 0.2 and 0.1, which suggest that all probabilities for these classes should not be discarded. Predictions for targets with a query-to-train distance below 0.03 should be considered unreliable, as these models do not encapsulate sufficient chemical space for a given query compound.

One way to address the influence of SE data with respect to the external model applicability domain is to make a comparison with and without SE training data for target models. This analysis was conducted for targets encompassing a good number (more than 1000) of inactives, which still require additional SE sampling of compounds to gain the 1:100 class ratio. 20 targets containing WOMBAT testing data fit this description. In 19 of the 20 cases, SE sampling improved the WOMBAT prevision performance (Table 3). Q14833, was the only target with decreased precision, which was assigned values of 0 due to the absence of active predictions caused by increased class imbalance.

**Table 3:** Influence of sphere excluded molecules for targets with 1000 or more confirmed inactives

Uniprot	Precision	Recall	SE Precision	SE Recall	∆Precision	∆Recall
O75116	0.006	0.960	0.012	0.960	0.006	0.000
P00374	0.187	0.982	0.520	0.968	0.333	−0.014
P00797	0.174	1.000	0.297	0.982	0.124	−0.018
P08172	0.091	0.912	0.307	0.829	0.216	−0.082
P08908	0.134	0.850	0.159	0.833	0.025	−0.017
P09237	0.013	0.991	0.046	0.981	0.033	−0.009
P11511	0.096	0.970	0.358	0.975	0.261	0.004
P25024	0.750	0.414	0.750	0.414	0.000	0.000
P28221	0.220	0.849	0.251	0.847	0.031	−0.002
P28222	0.225	0.859	0.282	0.839	0.058	−0.020
P31645	0.010	1.000	0.153	0.914	0.143	−0.086
P34913	0.146	0.980	0.191	0.975	0.045	−0.005
P34972	0.048	0.931	0.366	0.813	0.318	−0.117
P34995	0.026	0.935	0.232	0.761	0.206	−0.174
P41146	0.086	0.971	0.210	0.953	0.125	−0.018
P43115	0.090	0.743	0.700	0.700	0.610	−0.043
Q14833	0.004	0.500	0.000	0.000	−0.004	−0.500
Q15722	0.180	0.923	0.362	0.836	0.181	−0.086
Q99572	0.081	0.627	0.142	0.591	0.061	−0.036
Q99705	0.087	0.969	0.306	0.881	0.218	−0.089

Sphere Excluded molecules improves precision of the models at the cost of recall

### Comparison to an activity-only based model and other tools for target prediction

Predictions were generated for the compounds in the WOMBAT database using a model created solely on activity data from ChEMBL. The recall and precision results for the top-*k* ranking target positions for the predictions are shown in Table 4 and Additional file 1: Figure S1. The analysis exhibits the increase in recall and decrease in precision from 0.5 and 0.48 to 0.909 and 0.079 respectively, as the numbers of top-k ranking targets are increased from 3 to 180 positions. Table 4 shows that in order to achieve a comparable recall of 79.5 %, as achieved by precision thresholds, the activity-only model requires more than 54 ranked targets to be included. The corresponding precision value for a top-k position of 54 is approximately 14 %, considerably lower than the corresponding inactivity inclusive model precision of 24.3 %, indicating that methods including inactivity predict comparatively fewer false positives.

**Table 4:** Average precision and recall in the Top-*k* positions achieved for WOMBAT using the activity-only based model

Top-k positions	Recall (%)	Precision (%)
3	50.0	48.5
6	58.8	41.1
9	62.5	36.3
27	72.5	24.8
54	79.2	18.7
72	82.2	16.3
90	84.2	14.0
180	90.9	7.9

Increasing the number of top-*k* positions increases the recall and decreases the precision of the models

**Fig. 7:**
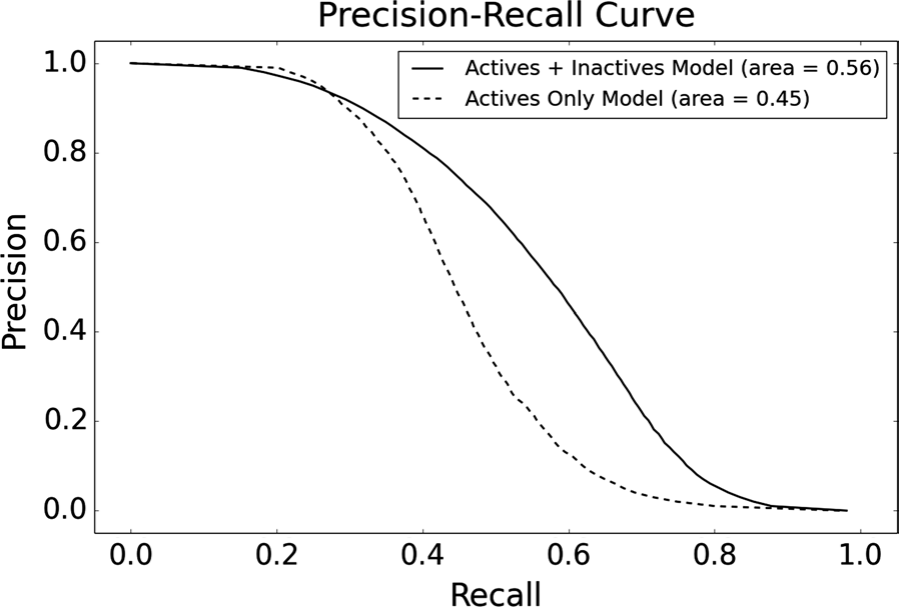
Precision-recall curve for the models including inactive data, and actives-only models. Including inactive data for models improves the recall and precision as shown by the AUC values for both plots, showing value for including inactive data when compared to models built exclusively on ChEMBL data

**Fig. 8:**
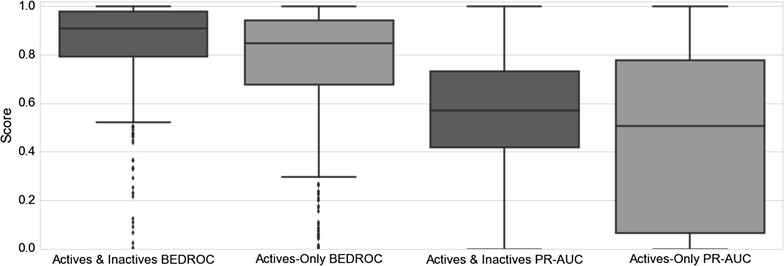
Boxplot of precision-recall curve AUC and BEDROC values for the models including inactive data, and actives-only models. Including inactive data for models improves the recall and precision AUC and BEDROC performance

Figure 7 illustrates the improved performance for models with inactivity data when considering averaged precision and recall curves for each method. Figure 8 demonstrates the boxplot precision-recall AUC metrics for individual models, with active and inactive models producing an average AUC value of 0.56, compared to actives-only models which produce an average AUC value of 0.45. Figure 8 also indicates the improved early recognition potential when including negative training instances, with the active and inactive models performing with an averaged BEDROC score of 0.85, compared to 0.76 obtained by active-only models. A Wilcoxon signed-rank test was conducted for the BEDROC and AUC scores, producing p values of 7.08e−08 and 4.96e−05 respectively, indicating the mean BEDROC and AUC values for the active and inactives model are statistically different from the mean BEDROC and AUC values for the actives-only model. When comparing the models built here to the Naïve Bayes method proposed by Koutsoukas et al. [39], in order to achieve a recall of between 80 and 87 % as reported in this study, the author’s method requires top-*k* positions between 6 and 27 in order to achieve comparable recall performance. It is not possible to comment on differences in the precision obtained between the prediction tools, as the authors did not report precision values.

## Experimental

### Model workflow

**Fig. 9:**
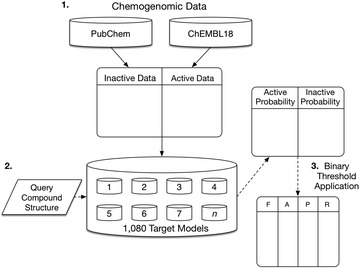
Flow of training and query data through the prediction approach described in this work. **1.** The PubChem inactives and ChEMBL actives used to train the Bernoulli Naïve Bayes classifier. **2.** The query data is imported and classification is performed producing probability scores. **3.** If binary predictions are required, class-specific cut-off thresholds can be applied. F, A, P and R represent the F1, accuracy, precision and recall metrics used to calculate thresholds. *Block arrows* represent the flow of training data, while dashed arrows represent query fingerprint or prediction score data flow

The overall flow of data for the realised tool is depicted in Fig. 9. Data flow can be split into three main sections; Step 1 is concerned with the extraction of data from the PubChem and ChEMBL18 repositories for use in the models. Step 2 involves the training of the machine learning methods using the bioactivity training data on a per class basis. Input compound queries are converted into 2048 bit Morgan fingerprints using Rdkit [52], imported into NumPy vectors, and passed to the BernoulliNB Scikit-learn module. For future model usage, the models are exported to file using the ‘cPickle’ Python library, enabling quick access and compact storage of the data. Step 3 shows the optional application of the binary decision thresholds, as the software allows either output binary predictions or the raw probabilistic output of the predictions.

### Active data set

ChEMBL [45] (Version 18) was used to construct the active bioactivity training data set. This version of the database encompasses over 12 million manually curated bioactivites, spanning over more than 9000 protein targets and over 1 million distinct compounds. Bioactivities were extracted for activity values (IC50/EC50/K_i_/K_d_) of 10 μM or lower, with a CONFIDENCE_SCORE of 5 or greater for ‘binding’ or ‘functional’ human protein assays. The 10 μM cut-off for activity specified here is in accordance with the method employed in the study of Koutsoukas et al. [39], representing both marginally and highly active compounds. Finally, ChEMBL polymer_flag and inorganic_type flags were included to ensure only suitable compounds qualified for the resulting training set.

After extraction, compound SMILES were standardized using the ChemAxon Command-Line Standardizer [53], with options “Remove Fragment” (keep largest), “Neutralize”, “RemoveExplicitH”, “Clean2d”, “Mesomerize”, and “Tautomerize” specified in the configuration file. The standardized canonical SMILES were then filtered for small or large compounds (100 Da < MW < 900 Da) and checked for duplicate ligand structures to ensure only one set of protein–ligand pairs were retained. Protein classes comprising less than 10 compounds were discarded since they did not comprise sufficient amounts of training data to learn from.

The complete active data set encompasses over 295,000 bioactivities covering 1080 protein classes. A large proportion of the classes included in the model are enzymes and membrane receptors, encompassing 57 and 17 % of the data respectively. A considerable percentage of compounds (33 %) were annotated for more than one target.

### Effect of confidence score on activity data sets

The allocation of a confidence score forms part of the manual curation processes that is applied to ChEMBL, and is used to describe the assay-to-target relationships represented in the database. Recent studies utilizing the ChEMBL database as a bioactivity data source have specified stringent confidence scores of 8 or 9 [39, 46, 51], and in order to explore this parameter more fully we sought to analyse the differences in the data sets that are extracted from the ChEMBL database when a variety of confidence scores are specified (Table 5). This parameter is unexplored throughout literature and its influence on resulting training-sets for target prediction is relatively undocumented.

**Table 5:** Descriptions used for the ChEMBL confidence scores

Confidence score	Description
0	Default value—target assignment has yet to be curated
1	Target assigned is non-molecular
3	Target assigned is molecular non-protein target
4	Multiple homologous protein targets may be assigned
5	Multiple direct protein targets may be assigned
6	Homologous protein complex subunits assigned
7	Direct protein complex subunits assigned
8	Homologous single protein target assigned
9	Direct single protein target assigned

A confidence score of 5 was used in this study to ensure that multi-domain proteins are not excluded from training data. Confidence scores of 9 or 8 are frequently used which specify homologous or direct proteins to be assigneds

Ranges of confidence scores (between ≥5 and ≥9) were applied to the ChEMBL18 database and the structure of the data sets extracted and the number of classes were calculated (Additional file 1: Table S2). A confidence score of 5 gives 1080 target activity classes containing 10 or more data points, with the number of classes decreasing with higher scores. A total of 306 classes (28.4 % of the data) were removed between the scores 5 to 9. The sharpest increase in mean and median class size and the total number of classes are observed between the scores 9 to 8, increasing from 774 to 959, respectively. A confidence score of 5 was applied in this study, since this threshold enables data with “multiple direct protein targets”, providing a suitable trade-off between confidence of the reliability of the data and training data size. Higher score values exclude “Homologous multi-domain protein associations” (scores ≥6) and “Direct multi-domain protein associations” (scores ≥7), such as the GABA receptors.

### Inactive data set

**Fig. 10:**
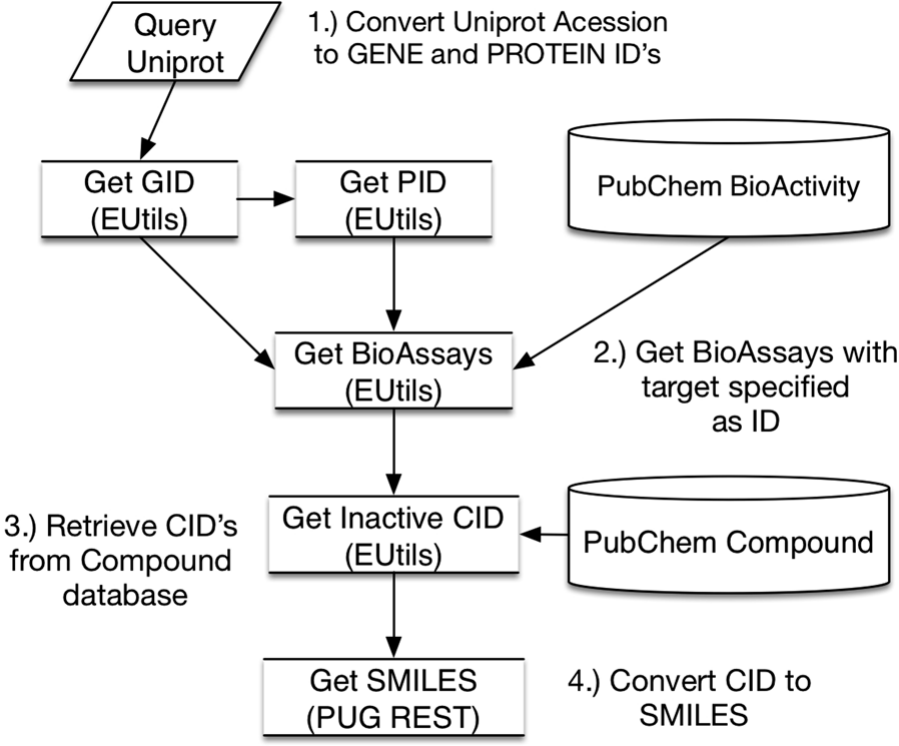
Flow chart for PubChem inactives retrieval. The process involves translating from the UniProt target name, to the Gene ID (GID) and (potentially) a Protein ID (PID), to identify BioAssay ID’s (AID) with annotated inactive Compound ID’s (CID). The “PUBCHEM ACTIVITY OUTCOME” field, completed by the publishing author or an equivalent expert in the field, is used to identify inactive compounds. EUtils is used to extract all assays and respective bioactivity outcome annotations to obtain chemicals declared to be “inactive” against a given target from an assay. PubChem PUG REST is used for the subsequent batch translation of CID’s to SMILES

For the purpose of this study, the PubChem Compound and PubChem BioAssay databases were mined for inactive compounds, and an automated extraction pipeline was developed to obtain inactive compound-ligand pairs from the PubChem database using the Entrez Programming Utilities (EUtils) [54, 55]. The EUtils are a set of server-side programs that provide an interface to query the various databases at NCBI [55]. Contrary to ChEMBL, the data contained in PubChem BioAssay are not manually curated, and the metadata for targets that are listed on assay records are as provided by the submitting researchers. The flowchart for the script is outlined in Fig. 10, which involves both ‘ESearch’ (to query the gene and protein databases) and ‘ELink’ (to connect to the assay database) procedures to obtain all Gene IDs (GIDs) and Protein IDs (PIDs) for a given UniProt Accession (including any IDs that are not included in UniProt) [56]. The script retrieves assays for the GIDs and PIDs from the BioAssay database and links to the Compound database to identify Compound IDs (CIDs) annotated by the submitting researcher as inactive when assayed in the studies. In a final step, the pipeline retrieves the SMILES for the compounds using the PubChem Power User Gateway (PUG) REST service [57], a protocol preferred for the final step as it is optimized for the bulk retrieval of data [58]. This methodology ensures that regardless whether an assay has a UniProt Accession, GenPept ID, RefSeq ID or GeneID etc. specified for an assay, we were able to retrieve all bioactivites with a defined target the same as indicated by the UniProt accession.

In order to ensure only appropriate molecules are retained in the inactive data set, RDKit was used to flag structures without a carbon molecule and molecules containing unwanted heavy metals, such as Lithium, Beryllium, Boron, Fluorine, Sodium, Aluminum, Silicon, Argon, Titanium, Iron, Zinc and Bromine. The filtered SMILES were subject to the same ChemAxon standardization filtering as the active ChEMBL bioactivity data set, i.e. duplicate molecules were removed, including compounds with a MW of below 100 or above 900 Da.

The PubChem inactive data set includes more than 194 million protein–ligand pairs spanning approximately 648,000 distinct compound structures (Additional file 1: Table S3). More than 647,900 of these compounds were annotated for two or more protein targets, producing a well-populated matrix of inactive-compound annotations. The annotation overlap for target-annotated chemistry is due to PubChem being initially established to be the storage site for automated high-throughput biological assays testing standardized libraries of compounds [58]. Secondly, it was also the central intention for the National Institutes of Health (NIH) Molecular Libraries program to have many of the laboratories test the same compounds on a wide range of targets, in an attempt to create a useful repository for mining in the future [59, 60].

### Sphere exclusion for putative inactive sampling

The data extracted from PubChem contains a very large variance in the number of inactives extracted for each of the targets, with some encompassing little or no experimental inactives. Mixing experimental inactives with putative inactives would alleviate this issue, at the cost of producing models that are no longer trained solely on confirmed evidence of inactivity. Although this caveat may remove the benefit of predicting on truly inactive data from the current state of the models, refraining from any additional sampling would result in overtly imbalanced training data for 480 targets, which would otherwise require removal.

**Fig. 11:**
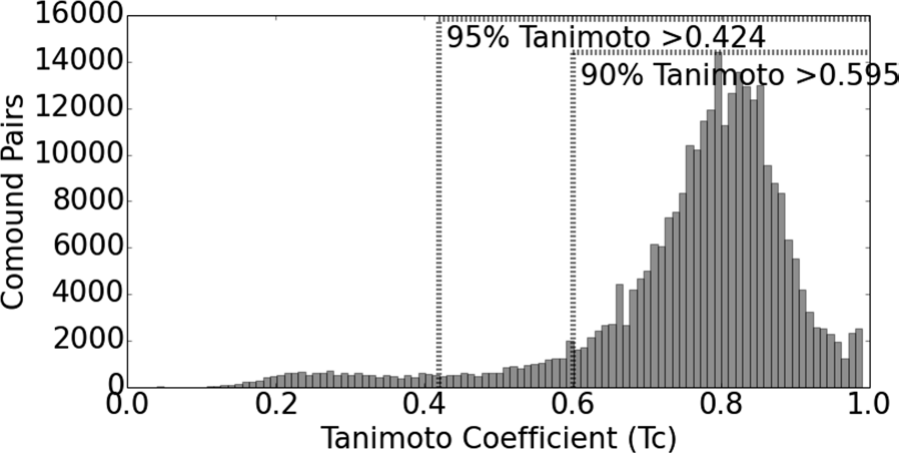
Distribution of Tanimoto coefficient (Tc) values for nearest neighbours for specific targets. A Tc value of 0.595 encompasses 90 % of the compound ligand pairs observed, while a value of 0.424 encapsulates 95 % of the compound pairs. Hence, a Tc value of 0.424 was employed as the exclusion radius to sample suitable ‘presumed inactive’ compounds

A sphere exclusion (SE) algorithm was utilized to randomly sample ‘presumed inactive’ compounds from a pool of ChEMBL compounds using a dissimilarity radius from known actives. Figure 11 shows a plot of the nearest neighbours for the classes from the active data set. A Tanimoto coefficient (Tc) of 0.424 was used as the radius of the exclusion sphere as it encompassed 95 % of the active neighbours. This value was considered a suitable threshold for the exclusion radius as defined by the value that encapsulates a large proportion of the similarity of the active data points, whilst also not requiring such dissimilar compounds as to select strange or undesirable chemistry. The putative inactive selection method proposed here is similar to the diversity selection algorithms proposed by Hudson et al. [61] and Gobbi et al. [62] although the SE algorithm proposed here is employed as a method of repeatedly sampling from outside an exclusion sphere for a required number of times until a specified number of presumed inactive compounds are selected, rather than a method for selecting diverse compounds.

The procedure was applied to 480 target classes that consist of few or no inactive compounds which produce an active-inactive ratio smaller than 1:100. This process sampled approximately 11 million additional inactives for the required targets. For situations with targets with very high numbers of inactive compounds, undersampling was performed through the randomised removal of instances from the inactive class to accomplish the desired ratio. The complete data set of 206,559,765 ligand-target pairs from both the active and inactive classes is available for use as a benchmark data set (see Additional file 1).

### Chemical descriptors

RDKit [52] was used to generate hashed ECFP_4 circular Morgan fingerprints [63] with a 2048 bit length. ECFP fingerprints were selected as they have previously been shown to be successful when attempting to capture relevant molecular information for in silico bioactivity prediction [39, 64].

## Conclusions

We have implemented a method for the extraction of inactive compounds from the PubChem repository. The application of a sphere exclusion algorithm enabled the oversampling of additional inactive compounds for targets with insufficient number of inactive compounds.

The realised target prediction protocol has been packaged and available for download at https://github.com/lhm30/PIDGIN.

The results from the internal and external validation of the tool show differing performance between the breadth of models. Recall and precision performance is influenced significantly on the underlying intraclass similarity and the number of compounds for a target. During internal validation, sphere exclusion models perform better in comparison to PubChem inactive classes, due to the dissimilarity requirement between the active compounds and SE inactive compounds.

The external performance of the models showed a considerable drop in precision, which may be exaggerated due to the absence of inactivity information held within the WOMBAT data set. Applications of target-specific thresholds exhibited a trade-off between recall and precision and indicate that different metrics for thresholds should be used for different applications of the target prediction tool. The precision threshold gleans the highest F-Score performance, producing a close balance between the precision and recall (0.5 and 0.48) for the classes. In comparison, the recall threshold can be applied to generate thresholds when predictions require high recall without the concern for the cost of sacrificing precision.

A distance-based analysis of the applicability domain (AD) for WOMBAT compounds showed that the reliability of the predictions from the models improved with the increasing similarity between the training and WOMBAT test set. The AD analysis indicated that an average Tc cut-off distance between test and train of 0.3 could be incorporated into the predictions of the classes in the future, to give insight into the degree of confidence for the probabilities generated by the model.

A comparison between the inactivity-inclusive models and the activity-only based approach showed the benefit in including negative bioactivity data when building the target prediction models with statistical significance. The ability to take into account the features that contribute and counteract bioactivity, combined with the ability to create individual target models, results in superior precision, recall, precision-recall AUC and BEDROC values when compared to models trained solely on activity data.

## Methods

### Model training using Bernoulli the Naïve Bayes classifier implemented in Scikit-learn

The Naïve Bayes algorithm was selected due to its basic implementation and demonstrated ability to perform in a variety of target prediction settings [28, 39, 40]. The specific classification algorithm of choice for this study is the Bernoulli Naïve Bayes algorithm, due to the ability of the algorithm to interpret the binary bit string features used to describe compound inputs. In comparison to other methods, this algorithm is also capable of maintaining its predictive power for highly imbalanced datasets, which is particularly beneficial when attempting to utilise large numbers of negative instances in training data [65, 66]. For example, it has been shown that enlarged negative training set sizes hinder the recall performance of the SMO, Random Forest, Ibk and J48 algorithms [67]. The preferable ratio of active to inactive compounds for these methods was found to be only around 1:9, a significant decrease from the 1:100 ratio envisaged for this study. Such algorithms would therefore require large scale undersampling of inactives data points to obtain acceptable performance, thus sacrificing the coverage of inactivity space.

In the target prediction context, an input molecule can be viewed as a vector of chemical features ***x*** = {*F*_1_,…, F_n_}, described by the fingerprints used to define a compound in relation to a target. Equation 1 shows the Bayes’ theorem, underpinning Bayesian models. This equation returns the ‘conditional probability’ or *p*(*C*|***x***), an evaluation of the certainty about a compound belong to class *C* [68].

1 $$p(C|{\mathbf{x}}) = \frac{{p(C)p({\mathbf{x}}|C)}}{{p({\mathbf{x}})}}$$

The likelihood function for Bernoulli Naïve Bayes is based on Eq. 2, which represents how likely a query compound ***x*** = *{F*_*1*_^*Test*^*, …, F*_*n*_^*Test*^*}* exhibits activity against a given target *C*. The *BernoulliNB* class from the Scikit-learn [69] library was employed to implement the Bernoulli Naïve Bayes algorithm.

2 $$P\left( {F_{1}^{Test} , \ldots ,F_{n}^{Test} |C} \right) = \sum\nolimits_{i = 1}^{n} {P(F_{i}^{Train} |C)F_{i}^{Test} + (1 - P(F_{i}^{Train} |C))(1 - F_{i}^{Test} )}$$

This algorithm explicitly penalizes the non-occurrence of a feature *i* that is indicative of activity class *C* [68]. For bioactivity prediction, this is recognized as the differential ability for the treatment of negative evidence within the fingerprints, i.e. a 0-bit being interpreted as the absence of an atom environment feature in a molecule [70]. This allows the models to explicitly identify trends including the absence of features within molecules.

### Internal model evaluation of the models

The StratifiedKFold function from Scikit-learn was used to split the data into train and test sets using fivefolds, while preserving the percentage of samples for each active and inactive class. For each of the *N* folds, the performance of the model can be measured in terms of precision and recall which can be presented as:

$${\text{Precision }} = \frac{TP}{TP + FP}\quad {\text{Recall }} = \frac{TP}{TP + FN}$$

where TP denotes true-positives and FP denotes false-positives. The precision and recall for each of the folds are averaged to give an overall metric for a particular class. This is repeated for all the classes in the data set.

### Target-specific activity threshold generation

Target-specific activity threshold values were calculated and employed to weight the learner algorithm to avoid the degenerate situation that can arise from class imbalance. Such thresholds allow the models to more precisely generate binary predictions based on a calculated probability of activity, i.e. activity if *Pa* ≥ threshold.

The procedure used to generate thresholds is founded upon on the methodology employed by Drakakis et al. [71] and requires three steps. First, the training data for each target is split into fivefolds. For each of the folds, probabilities are generated for the test compounds after training. Next, a range of threshold decision boundaries ganging between 0.1 and 1.0 (with increments of 0.001) are applied to the raw probability scores for the folds to generate binary predictions. The performances of each threshold are calculated independently using precision, recall, accuracy, and the F1-score as performance metrics. The Accuracy and F1 metrics are denoted as:

$${\text{Accuracy}}(y,\hat{y}) = \frac{1}{{n_{\text{samples}} }}\sum\limits_{i = 0}^{{n_{\text{samples}} - 1}} {1(\hat{y}_{i} = {\text{y}}_{i} )} \quad F_{1} = 2\cdot\frac{{{\text{precision}}\cdot{\text{recall}}}}{{{\text{precision}} + {\text{recall}}}}$$

Finally, the overall performance of each threshold is averaged for each of the folds, and the threshold with the optimal performance over the five-fold yields the optimum decision threshold for that specific target. This process is conducted for each of the performance metrics to produce metric-specific thresholds which will be discussed in more detail in the results section.

### External model evaluation

The WOMBAT [72] database contains compounds that are not annotated in the PubChem and ChEMBL databases. A data set of annotated compounds was extracted from the WOMBAT database (version 2011.1) considering activities for Ki, IC50, Kd or EC50 with values of 10 μM or smaller. Bioactivites also contained in the ChEMBL or PubChem training sets (identified as Tanimoto values between structures of 1.0) were removed, resulting in the removal of 3624 WOMBAT compounds. In total 65,123 active compounds were retained for testing, comprising 418 protein target classes. Performance of the models was measured using precision and recall after application of the various class-specific binary thresholds (previously calculated) for each target.

### Evaluation of class-specific thresholds

A practical way in which to systematically assess the value in applying the different decision thresholds has been devised. This system uses a ratio scoring system to demonstrate the performance enhancement when using the class-specific thresholds against cases when no threshold is used. The enrichment ratio function (*R*_*Score*_) is defined as:

$$R_{Score} \, = { \log }_{2} \left( {\frac{{Score_{\text{Threshold}} }}{{Score_{\text{No Threshold}} }}} \right)$$

Here, Score_Threshold_ represents the F_1_ performance score obtained for a class when applying a decision threshold. This value is divided by Score_No Threshold_, which is represented by the F_1_ performance score obtained without a threshold. The final step for completing the ratio *R* is to calculate the log base 2 of this value. A positive number from this function will denote a value in the application of a threshold (in terms of F_1_-Score), while a negative result will signify that there is value in the default selection of ‘activity if P*a* > P*i’*. Using this formula, enrichment ratios were calculated for the performances of the F_1_, mean Accuracy, Precision, and Recall decision boundaries. These functions are defined as:

$$\begin{aligned} R_{{F_{1} }} = \log_{2} \left( {\frac{{F_{{ 1_{\text{Threshold}} }} }}{{F_{{1_{{{\text{No}}{\kern 1pt} {\text{Threshold}}}} }} }}} \right)\quad R_{\text{Accuracy}} = \log_{2} \left( {\frac{{{\text{Accuracy}}_{\text{Threshold}} }}{{{\text{Accuracy}}_{\text{No Threshold}} }}} \right) \hfill \\ R_{\text{Precision}} = \log_{2} \left( {\frac{{{\text{Precision}}_{\text{Threshold}} }}{{{\text{Precision}}_{\text{No Threshold}} }}} \right)\quad R_{\text{Recall}} = \log_{2} \left( {\frac{{{\text{Recall}}_{\text{Threshold}} }}{{{\text{Recall}}_{\text{No Threshold}} }}} \right) \hfill \\ \end{aligned}$$

### Applicability domain estimation

A major problem regarding the practical applications of target prediction models is the unassessed reliability of the predictions [73]. The function of the applicability domain (AD) is to indicate when the assumptions made by a model are fulfilled and which input chemicals are reliably appropriate for the models [8, 74, 75]. A distance-based AD approach was employed to analyse the distances between a query compound to the nearest neighbour in the training data [75]. The distance from the test and training set is then cross-referenced with the probability score for activity for active compounds from WOMBAT, giving a measurement for the active prediction performance of the models.

### Construction and performance evaluation of a model trained on activity only

A single model was trained based on activity data from ChEMBL exclusively. More specifically, a Bernoulli Naïve Bayes algorithm was trained on the fingerprints from the target-compound associations from the 1080 targets extracted from the ChEMBL dataset. Similarly to the previous models, 2048 bit Morgan fingerprints were imported into Scikit-learn class, giving a model containing almost 300 thousand active compounds. The probability scores generated by this model produce predictions that a compound is active for a given target when considering the probability that the molecule is also active for the other targets.

## Additional file

10.1186/s13321-015-0098-y Supplementary data describing the scaffold composition, size of models and performance of actives-only ranking positions.
