# Supplementary material for: Target prediction utilising negative bioactivity data covering large chemical space
Source: J Cheminform. 2015 Oct 24;7:51. doi: 10.1186/s13321-015-0098-y (PMC4619454; doi:10.1186/s13321-015-0098-y)
Supplement: Supplementary file 1 — 10.1186/s13321-015-0098-y Supplementary data describing the scaffold composition, size of models and performance of actives-only ranking positions. [file 13321_2015_98_MOESM1_ESM.docx]

# Supporting Information

| **ChEMBL** | | | | |
| --- | --- | --- | --- | --- |
| **Rank** | **Count** | **SMILES** | **Structure** | |
| **1** | 2756 | c1ccccc1 |  | |
| **2** | 505 | c1ccc(-c2ccccc2)cc1 |  | |
| **3** | 422 | c1ccc(Oc2ccccc2)cc1 |  | |
| **4** | 346 | c1cc2ccccc2[nH]1 |  | |
| **5** | 306 | c1ccc(Nc2ncnc3ccccc32)cc1 |  | |
| **6** | 263 | c1ccc2ccccc2c1 |  | |
| **7** | 241 | O=S(=O)(Nc1ccccc1)c1ccccc1 |  | |
| **8** | 216 | c1cn(Cc2ccccc2)c2ccccc12 |  | |
| **9** | 207 | c1ccc(COc2ccccc2)cc1 |  | |
| **10** | 193 | c1ccc(C2CC3CCC(C2)N3)cc1 |  | |
| **PubChem** | | | | |
| **Rank** | **Count** | **SMILES** | | **Structure** |
| **1** | 14,163 | c1ccccc1 | |  |
| **2** | 3,285 | O=C(Nc1ccccc1)c1ccccc1 | |  |
| **3** | 1,669 | O=S(=O)(Nc1ccccc1)c1ccccc1 | |  |
| **4** | 1,567 | O=C(COc1ccccc1)Nc1ccccc1 | |  |
| **5** | 1,268 | c1ccncc1 | |  |
| **6** | 1,162 | c1ccc2ncccc2c1 | |  |
| **7** | 1,036 | O=C(CNc1ccccc1)Nc1ccccc1 | |  |
| **8** | 979 | O=C1NCCCCNCCOc2ccccc21 | |  |
| **9** | 930 | c1cc2ccccc2[nH]1 | |  |
| **10** | 787 | c1ccsc1 | |  |

**Table S1.** A Murcko scaffold decomposition analysis of the ChEMBL and PubChem training sets. There is overlap within the top 10 ranked Murcko scaffolds generated for the ChEMBL and PubChem datasets. The benzene ring was calculated to be the number one ranked scaffold structure in both. Many of the other scaffolds show close resemblance between both datasets, with the Diphenylether and N-Phenyl-Benzenesulfonamide structures present in both data sets.

|  | | **Class Size** | | | |
| --- | --- | --- | --- | --- | --- |
| **Confidence Score** | **Count of classes** | **Mean** | **Median** | **Maximum** | **St. Deviation** |
| **≥ 5** | 1,080 | 274.04 | 85.5 | 3,423 | 460.21 |
| **≥ 6** | 1,041 | 276.63 | 79 | 3,423 | 457.46 |
| **≥ 7** | 1,025 | 265.86 | 74 | 3,393 | 447.21 |
| **≥ 8** | 959 | 264.98 | 61 | 3,289 | 435.79 |
| **≥ 9** | 774 | 179.39 | 25 | 2,398 | 280.49 |

**Table S2.** An analysis of the resulting datasets extracted from ChEMBL after application of various confidence scores. Increasing the confidence score in ChEMBL removes large amounts of classes from the resulting data sets. Class counts are calculated from targets comprising greater or equal to 10 compounds with associated active bioactivites.

| **NAME** | **UNIPROT** | **ACTIVES** | **INACTIVES** | **RATIO** |
| --- | --- | --- | --- | --- |
| Krueppel-like factor 5 | A2TJX0 | 37 | 287634 | 7774 |
| 5-hydroxytryptamine receptor 3E | A5X5Y0 | 148 | 15 | 0 |
| Serine/threonine-protein kinase Sgk1 | O00141 | 15 | 327090 | 21806 |
| Thymidine kinase 2, mitochondrial | O00142 | 14 | 14 | 1 |
| Metabotropic glutamate receptor 8 | O00222 | 10 | 6 | 1 |
| Bone morphogenetic protein receptor type-1B | O00238 | 16 | 335333 | 20958 |
| Menin | O00255 | 15 | 419244 | 27950 |
| Cell division cycle 7-related protein kinase | O00311 | 495 | 286 | 1 |
| Phosphatidylinositol 4,5-bisphosphate 3-kinase catalytic subunit delta isoform | O00329 | 752 | 189533 | 252 |
| cGMP-dependent 3',5'-cyclic phosphodiesterase | O00408 | 118 | 8 | 0 |
| Eukaryotic elongation factor 2 kinase | O00418 | 23 | 64805 | 2818 |
| Serine/threonine-protein kinase PLK4 | O00444 | 236 | 30 | 0 |
| Nuclear receptor subfamily 5 group A member 2 | O00482 | 29 | 356828 | 12304 |
| Fatty-acid amide hydrolase 1 | O00519 | 521 | 5 | 0 |
| Cocaine esterase | O00748 | 81 | 0 | 0 |
| Phosphatidylinositol 4-phosphate 3-kinase C2 domain-containing subunit beta | O00750 | 13 | 64454 | 4958 |
| Acetyl-CoA carboxylase 2 | O00763 | 278 | 1 | 0 |
| Acyl-CoA desaturase | O00767 | 253 | 1 | 0 |
| Citron Rho-interacting kinase | O14578 | 10 | 37 | 4 |
| Disintegrin and metalloproteinase domain-containing protein 10 | O14672 | 109 | 362657 | 3327 |
| Prostaglandin E synthase | O14684 | 246 | 255475 | 1039 |
| Apoptotic protease-activating factor 1 | O14727 | 15 | 376241 | 25083 |
| Telomerase reverse transcriptase | O14746 | 116 | 403376 | 3477 |
| Serine/threonine-protein kinase Chk1 | O14757 | 1290 | 503746 | 391 |
| Free fatty acid receptor 1 | O14842 | 332 | 223867 | 674 |
| Inhibitor of nuclear factor kappa-B kinase subunit beta | O14920 | 565 | 400151 | 708 |
| Phospholipase D2 | O14939 | 72 | 421704 | 5857 |
| Aurora kinase A | O14965 | 1266 | 432323 | 341 |
| Cyclin-G-associated kinase | O14976 | 19 | 341878 | 17994 |
| Serine/threonine-protein kinase DCLK1 | O15075 | 17 | 0 | 0 |
| Inhibitor of nuclear factor kappa-B kinase subunit alpha | O15111 | 149 | 422232 | 2834 |
| 1-acyl-sn-glycerol-3-phosphate acyltransferase beta | O15120 | 77 | 0 | 0 |
| Ephrin type-B receptor 6 | O15197 | 11 | 52 | 5 |
| Mitogen-activated protein kinase 13 | O15264 | 213 | 48 | 0 |
| Histone deacetylase 3 | O15379 | 438 | 420291 | 960 |
| Baculoviral IAP repeat-containing protein 5 | O15392 | 20 | 339629 | 16981 |
| Group 10 secretory phospholipase A2 | O15496 | 21 | 0 | 0 |
| 3-phosphoinositide-dependent protein kinase 1 | O15530 | 251 | 402256 | 1603 |
| Free fatty acid receptor 2 | O15552 | 10 | 216337 | 21634 |
| Intermediate conductance calcium-activated potassium channel protein 4 | O15554 | 19 | 9 | 0 |
| Cytochrome P450 26A1 | O43174 | 93 | 7 | 0 |
| Motilin receptor | O43193 | 200 | 2 | 0 |
| Death-associated protein kinase 3 | O43293 | 260 | 64 | 0 |
| Mitogen-activated protein kinase kinase kinase 7 | O43318 | 57 | 414062 | 7264 |
| Receptor-interacting serine/threonine-protein kinase 2 | O43353 | 15 | 383961 | 25597 |
| Maltase-glucoamylase, intestinal | O43451 | 28 | 215192 | 7685 |
| Voltage-dependent T-type calcium channel subunit alpha-1G | O43497 | 85 | 48 | 1 |
| Potassium voltage-gated channel subfamily KQT member 3 | O43525 | 63 | 14 | 0 |
| Potassium voltage-gated channel subfamily KQT member 2 | O43526 | 86 | 44 | 1 |
| Carbonic anhydrase 12 | O43570 | 760 | 11 | 0 |
| Galanin receptor type 2 | O43603 | 12 | 139082 | 11590 |
| Orexin receptor type 1 | O43613 | 294 | 321241 | 1093 |
| Orexin receptor type 2 | O43614 | 305 | 202 | 1 |
| Dual specificity tyrosine-phosphorylation-regulated kinase 3 | O43781 | 111 | 0 | 0 |
| Aldo-keto reductase family 1 member B10 | O60218 | 43 | 0 | 0 |
| Transmembrane protease serine 11D | O60235 | 42 | 0 | 0 |
| NUAK family SNF1-like kinase 1 | O60285 | 12 | 268898 | 22408 |
| Lysine-specific histone demethylase 1A | O60341 | 10 | 6733 | 673 |
| Cyclin-T1 | O60563 | 80 | 383032 | 4788 |
| Toll-like receptor 2 | O60603 | 16 | 360667 | 22542 |
| Tyrosine-protein kinase JAK2 | O60674 | 931 | 406627 | 437 |
| ATP-binding cassette sub-family C member 9 | O60706 | 16 | 58 | 4 |
| Protein-S-isoprenylcysteine O-methyltransferase | O60725 | 69 | 189582 | 2748 |
| Potassium/sodium hyperpolarization-activated cyclic nucleotide-gated channel 1 | O60741 | 17 | 0 | 0 |
| Galanin receptor type 3 | O60755 | 77 | 351294 | 4562 |
| Hematopoietic prostaglandin D synthase | O60760 | 15 | 1 | 0 |
| Bromodomain-containing protein 4 | O60885 | 63 | 15 | 0 |
| Receptor activity-modifying protein 1 | O60894 | 20 | 4 | 0 |
| Cathepsin L2 | O60911 | 54 | 14 | 0 |
| Rho-associated protein kinase 2 | O75116 | 759 | 59556 | 78 |
| A disintegrin and metalloproteinase with thrombospondin motifs 4 | O75173 | 117 | 218019 | 1863 |
| Nuclear receptor corepressor 1 | O75376 | 20 | 428422 | 21421 |
| Nuclear receptor subfamily 1 group I member 2 | O75469 | 43 | 358598 | 8339 |
| Ribosomal protein S6 kinase alpha-5 | O75582 | 150 | 247710 | 1651 |
| Ribosomal protein S6 kinase alpha-4 | O75676 | 27 | 175891 | 6514 |
| Serine/threonine-protein kinase 16 | O75716 | 13 | 17 | 1 |
| Transient receptor potential cation channel subfamily A member 1 | O75762 | 88 | 27 | 0 |
| Gamma-aminobutyric acid type B receptor subunit 2 | O75899 | 13 | 64751 | 4981 |
| Diacylglycerol O-acyltransferase 1 | O75907 | 371 | 8 | 0 |
| Sterol O-acyltransferase 2 | O75908 | 92 | 1 | 0 |
| Serine/threonine-protein kinase PAK 3 | O75914 | 12 | 74 | 6 |
| cGMP-specific 3',5'-cyclic phosphodiesterase | O76074 | 937 | 322389 | 344 |
| High affinity cGMP-specific 3',5'-cyclic phosphodiesterase 9A | O76083 | 51 | 14 | 0 |
| Serine/threonine-protein kinase 10 | O94804 | 15 | 9 | 1 |
| Serine/threonine-protein kinase D3 | O94806 | 200 | 339794 | 1699 |
| G2/mitotic-specific cyclin-B2 | O95067 | 237 | 289 | 1 |
| Sphingosine 1-phosphate receptor 2 | O95136 | 73 | 228466 | 3130 |
| Voltage-dependent T-type calcium channel subunit alpha-1H | O95180 | 58 | 99576 | 1717 |
| High affinity cAMP-specific and IBMX-insensitive 3',5'-cyclic phosphodiesterase 8B | O95263 | 107 | 0 | 0 |
| 5-hydroxytryptamine receptor 3B | O95264 | 149 | 15 | 0 |
| Tankyrase-1 | O95271 | 68 | 314753 | 4629 |
| Urotensin-2 | O95399 | 26 | 10 | 0 |
| Tyrosyl-DNA phosphodiesterase 2 | O95551 | 41 | 372510 | 9086 |
| Neurotensin receptor type 2 | O95665 | 46 | 147 | 3 |
| Steroid hormone receptor ERR2 | O95718 | 24 | 370751 | 15448 |
| Geranylgeranyl pyrophosphate synthase | O95749 | 27 | 3 | 0 |
| Mitogen-activated protein kinase kinase kinase kinase 4 | O95819 | 350 | 326632 | 933 |
| Malonyl-CoA decarboxylase, mitochondrial | O95822 | 203 | 3 | 0 |
| Sphingosine 1-phosphate receptor 4 | O95977 | 241 | 230338 | 956 |
| Serine/threonine-protein kinase PAK 4 | O96013 | 126 | 304676 | 2418 |
| Serine/threonine-protein kinase Chk2 | O96017 | 311 | 423040 | 1360 |
| G1/S-specific cyclin-E2 | O96020 | 452 | 313 | 1 |
| L-lactate dehydrogenase A chain | P00338 | 22 | 65 | 3 |
| Dihydrofolate reductase | P00374 | 394 | 3745 | 10 |
| Coagulation factor XIII A chain | P00488 | 57 | 369483 | 6482 |
| Purine nucleoside phosphorylase | P00491 | 154 | 365058 | 2371 |
| Hypoxanthine-guanine phosphoribosyltransferase | P00492 | 22 | 5 | 0 |
| Tyrosine-protein kinase ABL1 | P00519 | 813 | 528730 | 650 |
| Epidermal growth factor receptor | P00533 | 2240 | 395109 | 176 |
| Prothrombin | P00734 | 2390 | 372850 | 156 |
| Complement C1r subcomponent | P00736 | 12 | 61952 | 5163 |
| Coagulation factor IX | P00740 | 131 | 214998 | 1641 |
| Coagulation factor X | P00742 | 3423 | 109 | 0 |
| Plasminogen | P00747 | 202 | 370682 | 1835 |
| Urokinase-type plasminogen activator | P00749 | 412 | 304232 | 738 |
| Tissue-type plasminogen activator | P00750 | 72 | 376996 | 5236 |
| Renin | P00797 | 1959 | 63939 | 33 |
| Adenosine deaminase | P00813 | 34 | 367770 | 10817 |
| Carbonic anhydrase 1 | P00915 | 1636 | 20 | 0 |
| Carbonic anhydrase 2 | P00918 | 2884 | 36 | 0 |
| Proto-oncogene c-Fos | P01100 | 23 | 433648 | 18854 |
| GTPase HRas | P01112 | 29 | 367679 | 12679 |
| Natriuretic peptides A | P01160 | 10 | 364327 | 36433 |
| Tumor necrosis factor | P01375 | 48 | 362667 | 7556 |
| Acetylcholine receptor subunit alpha | P02708 | 15 | 104 | 7 |
| Estrogen receptor | P03372 | 1183 | 448492 | 379 |
| Coagulation factor XI | P03951 | 40 | 219161 | 5479 |
| Plasma kallikrein | P03952 | 74 | 217816 | 2943 |
| Interstitial collagenase | P03956 | 1358 | 37 | 0 |
| 3-hydroxy-3-methylglutaryl-coenzyme A reductase | P04035 | 151 | 64796 | 429 |
| RAF proto-oncogene serine/threonine-protein kinase | P04049 | 300 | 502842 | 1676 |
| Phospholipase A2 | P04054 | 69 | 561 | 8 |
| Glucosylceramidase | P04062 | 68 | 305036 | 4486 |
| Tissue alpha-L-fucosidase | P04066 | 38 | 1 | 0 |
| Vitamin K-dependent protein C | P04070 | 17 | 214996 | 12647 |
| Apolipoprotein B-100 | P04114 | 42 | 451169 | 10742 |
| Glucocorticoid receptor | P04150 | 1456 | 477727 | 328 |
| HLA class II histocompatibility antigen, DRB1-1 beta chain | P04229 | 32 | 0 | 0 |
| HLA class II histocompatibility antigen gamma chain | P04233 | 11 | 300467 | 27315 |
| Sex hormone-binding globulin | P04278 | 53 | 341823 | 6449 |
| Tubulin beta-4A chain | P04350 | 25 | 156872 | 6275 |
| HLA class I histocompatibility antigen, A-3 alpha chain | P04439 | 16 | 0 | 0 |
| Receptor tyrosine-protein kinase erbB-2 | P04626 | 902 | 377378 | 418 |
| High affinity nerve growth factor receptor | P04629 | 314 | 368884 | 1175 |
| Calpain small subunit 1 | P04632 | 52 | 20 | 0 |
| Cellular tumor antigen p53 | P04637 | 90 | 573763 | 6375 |
| Thymidylate synthase | P04818 | 266 | 61 | 0 |
| Amyloid beta A4 protein | P05067 | 175 | 433253 | 2476 |
| Aldehyde dehydrogenase, mitochondrial | P05091 | 24 | 0 | 0 |
| Steroid 17-alpha-hydroxylase/17,20 lyase | P05093 | 252 | 76 | 0 |
| Integrin beta-3 | P05106 | 1793 | 370716 | 207 |
| Integrin beta-2 | P05107 | 305 | 113783 | 373 |
| Plasminogen activator inhibitor 1 | P05121 | 53 | 215540 | 4067 |
| Protein kinase C gamma type | P05129 | 147 | 411486 | 2799 |
| Myeloperoxidase | P05164 | 53 | 10 | 0 |
| Cytochrome P450 1A2 | P05177 | 10 | 3464 | 346 |
| Alkaline phosphatase, tissue-nonspecific isozyme | P05186 | 67 | 196194 | 2928 |
| Intercellular adhesion molecule 1 | P05362 | 405 | 366770 | 906 |
| Transcription factor AP-1 | P05412 | 61 | 448230 | 7348 |
| Fatty acid-binding protein, heart | P05413 | 12 | 1 | 0 |
| Integrin beta-1 | P05556 | 1114 | 346964 | 311 |
| Protein kinase C beta type | P05771 | 200 | 442158 | 2211 |
| Insulin receptor | P06213 | 318 | 374353 | 1177 |
| Tyrosine-protein kinase Lck | P06239 | 1190 | 429564 | 361 |
| Tyrosine-protein kinase Fyn | P06241 | 233 | 429298 | 1842 |
| Cholinesterase | P06276 | 464 | 81 | 0 |
| Progesterone receptor | P06401 | 1219 | 362920 | 298 |
| Cyclin-dependent kinase 1 | P06493 | 927 | 426447 | 460 |
| Eukaryotic translation initiation factor 4E | P06730 | 10 | 214887 | 21489 |
| Low affinity immunoglobulin epsilon Fc receptor | P06734 | 43 | 92340 | 2147 |
| Glycogen phosphorylase, liver form | P06737 | 342 | 0 | 0 |
| Nucleophosmin | P06748 | 25 | 419318 | 16773 |
| Integrin alpha-V | P06756 | 1026 | 363802 | 355 |
| Kallikrein-1 | P06870 | 33 | 0 | 0 |
| Prostate-specific antigen | P07288 | 24 | 48 | 2 |
| Tyrosine-protein kinase Fes/Fps | P07332 | 61 | 223627 | 3666 |
| Macrophage colony-stimulating factor 1 receptor | P07333 | 708 | 830 | 1 |
| Cathepsin D | P07339 | 442 | 195395 | 442 |
| Calpain-1 catalytic subunit | P07384 | 435 | 398998 | 917 |
| Tubulin beta chain | P07437 | 25 | 510418 | 20417 |
| Trypsin-1 | P07477 | 604 | 13 | 0 |
| Trypsin-2 | P07478 | 96 | 192944 | 2010 |
| Acetylcholine receptor subunit gamma | P07510 | 13 | 97 | 7 |
| Beta-2 adrenergic receptor | P07550 | 791 | 361135 | 457 |
| Cathepsin L1 | P07711 | 631 | 302279 | 479 |
| Cathepsin B | P07858 | 390 | 152106 | 390 |
| Heat shock protein HSP 90-alpha | P07900 | 262 | 432911 | 1652 |
| Tyrosine-protein kinase Yes | P07947 | 57 | 214998 | 3772 |
| Tyrosine-protein kinase Lyn | P07948 | 295 | 386172 | 1309 |
| Proto-oncogene tyrosine-protein kinase receptor Ret | P07949 | 367 | 385427 | 1050 |
| Insulin-like growth factor 1 receptor | P08069 | 729 | 449383 | 616 |
| Muscarinic acetylcholine receptor M2 | P08172 | 788 | 3587 | 5 |
| Muscarinic acetylcholine receptor M4 | P08173 | 235 | 357242 | 1520 |
| Multidrug resistance protein 1 | P08183 | 274 | 192949 | 704 |
| Mineralocorticoid receptor | P08235 | 307 | 300240 | 978 |
| Heat shock protein HSP 90-beta | P08238 | 245 | 421255 | 1719 |
| Neutrophil elastase | P08246 | 898 | 304253 | 339 |
| 72 kDa type IV collagenase | P08253 | 1931 | 364178 | 189 |
| Stromelysin-1 | P08254 | 1195 | 47 | 0 |
| Cathepsin G | P08311 | 50 | 338284 | 6766 |
| Neprilysin | P08473 | 232 | 25 | 0 |
| Integrin alpha-IIb | P08514 | 1007 | 369483 | 367 |
| Receptor-type tyrosine-protein phosphatase C | P08575 | 57 | 214303 | 3760 |
| Hepatocyte growth factor receptor | P08581 | 1242 | 380431 | 306 |
| Beta-1 adrenergic receptor | P08588 | 730 | 334663 | 458 |
| Tyrosine-protein kinase HCK | P08631 | 192 | 379042 | 1974 |
| Integrin alpha-5 | P08648 | 109 | 330028 | 3028 |
| Coagulation factor VII | P08709 | 417 | 117 | 0 |
| Steryl-sulfatase | P08842 | 153 | 8 | 0 |
| 5-hydroxytryptamine receptor 1A | P08908 | 2168 | 189541 | 87 |
| Muscarinic acetylcholine receptor M5 | P08912 | 142 | 357194 | 2515 |
| Alpha-2A adrenergic receptor | P08913 | 379 | 337993 | 892 |
| Proto-oncogene tyrosine-protein kinase ROS | P08922 | 117 | 352750 | 3015 |
| Glutathione S-transferase P | P09211 | 10 | 51 | 5 |
| Matrilysin | P09237 | 191 | 5280 | 28 |
| Fructose-1,6-bisphosphatase 1 | P09467 | 205 | 82161 | 401 |
| Platelet-derived growth factor receptor beta | P09619 | 676 | 391301 | 579 |
| Pro-cathepsin H | P09668 | 13 | 0 | 0 |
| Tyrosine-protein kinase Fgr | P09769 | 22 | 412880 | 18767 |
| Complement C1s subcomponent | P09871 | 73 | 61953 | 849 |
| Poly [ADP-ribose] polymerase 1 | P09874 | 1105 | 419936 | 380 |
| Arachidonate 5-lipoxygenase | P09917 | 846 | 90655 | 107 |
| Furin | P09958 | 63 | 352224 | 5591 |
| Leukotriene A-4 hydrolase | P09960 | 360 | 12 | 0 |
| Cytosolic phospholipase A2 beta | P0C869 | 22 | 1 | 0 |
| Pepsin A-5 | P0DJD9 | 15 | 0 | 0 |
| Granzyme B | P10144 | 19 | 332985 | 17526 |
| Interleukin-8 | P10145 | 17 | 300795 | 17694 |
| Androgen receptor | P10275 | 1025 | 448873 | 438 |
| Retinoic acid receptor alpha | P10276 | 106 | 366727 | 3460 |
| Acrosin | P10323 | 24 | 0 | 0 |
| Apoptosis regulator Bcl-2 | P10415 | 257 | 456298 | 1775 |
| Lysosomal protective protein | P10619 | 29 | 38 | 1 |
| Microtubule-associated protein tau | P10636 | 25 | 435949 | 17438 |
| Mast/stem cell growth factor receptor Kit | P10721 | 632 | 372968 | 590 |
| Retinoic acid receptor beta | P10826 | 157 | 358105 | 2281 |
| Thyroid hormone receptor alpha | P10827 | 163 | 362829 | 2226 |
| Thyroid hormone receptor beta | P10828 | 261 | 269603 | 1033 |
| Phenylethanolamine N-methyltransferase | P11086 | 61 | 0 | 0 |
| Glycogen phosphorylase, muscle form | P11217 | 71 | 0 | 0 |
| Muscarinic acetylcholine receptor M1 | P11229 | 780 | 355448 | 456 |
| Acetylcholine receptor subunit beta | P11230 | 13 | 93 | 7 |
| Breakpoint cluster region protein | P11274 | 156 | 510252 | 3271 |
| Serine/threonine-protein kinase pim-1 | P11309 | 704 | 380755 | 541 |
| Fibroblast growth factor receptor 1 | P11362 | 594 | 372561 | 627 |
| DNA topoisomerase 1 | P11387 | 153 | 444274 | 2904 |
| Vitamin D3 receptor | P11473 | 102 | 426689 | 4183 |
| Steroid hormone receptor ERR1 | P11474 | 82 | 376790 | 4595 |
| Aromatase | P11511 | 712 | 5549 | 8 |
| Cholesteryl ester transfer protein | P11597 | 404 | 1 | 0 |
| Cyclin-dependent kinase 4 | P11802 | 644 | 416898 | 647 |
| Inosine-5'-monophosphate dehydrogenase 2 | P12268 | 417 | 348466 | 836 |
| Bone morphogenetic protein 4 | P12644 | 13 | 336444 | 25880 |
| Angiotensin-converting enzyme | P12821 | 306 | 20 | 0 |
| Proto-oncogene tyrosine-protein kinase Src | P12931 | 1467 | 495528 | 338 |
| Bone morphogenetic protein 1 | P13497 | 297 | 6 | 0 |
| C-C motif chemokine 2 | P13500 | 22 | 91 | 4 |
| C-C motif chemokine 5 | P13501 | 27 | 77 | 3 |
| Cystic fibrosis transmembrane conductance regulator | P13569 | 68 | 379656 | 5583 |
| Integrin alpha-4 | P13612 | 1226 | 347340 | 283 |
| Retinoic acid receptor gamma | P13631 | 135 | 404182 | 2994 |
| Tissue factor | P13726 | 226 | 130 | 1 |
| Glycogen [starch] synthase, muscle | P13807 | 14 | 302759 | 21626 |
| Sodium/glucose cotransporter 1 | P13866 | 103 | 239808 | 2328 |
| Beta-3 adrenergic receptor | P13945 | 1105 | 347239 | 314 |
| 3 beta-hydroxysteroid dehydrogenase/Delta 5-->4-isomerase type 1 | P14060 | 13 | 0 | 0 |
| Estradiol 17-beta-dehydrogenase 1 | P14061 | 194 | 140 | 1 |
| Cathepsin E | P14091 | 32 | 127921 | 3998 |
| Macrophage migration inhibitory factor | P14174 | 28 | 419777 | 14992 |
| Perforin-1 | P14222 | 12 | 0 | 0 |
| Farnesyl pyrophosphate synthase | P14324 | 99 | 31 | 0 |
| Sucrase-isomaltase, intestinal | P14410 | 25 | 64746 | 2590 |
| D(2) dopamine receptor | P14416 | 2755 | 401225 | 146 |
| Phospholipase A2, membrane associated | P14555 | 173 | 146594 | 847 |
| Pyruvate kinase PKM | P14618 | 33 | 261958 | 7938 |
| G2/mitotic-specific cyclin-B1 | P14635 | 337 | 402195 | 1193 |
| Tyrosinase | P14679 | 10 | 1 | 0 |
| Matrix metalloproteinase-9 | P14780 | 1641 | 528 | 0 |
| Gamma-aminobutyric acid receptor subunit alpha-1 | P14867 | 594 | 33 | 0 |
| Indoleamine 2,3-dioxygenase 1 | P14902 | 54 | 73 | 1 |
| D-amino-acid oxidase | P14920 | 75 | 2 | 0 |
| Serine/threonine-protein kinase B-raf | P15056 | 408 | 452006 | 1108 |
| Carboxypeptidase B | P15086 | 19 | 0 | 0 |
| Fatty acid-binding protein, adipocyte | P15090 | 48 | 356688 | 7431 |
| Aldose reductase | P15121 | 408 | 1 | 0 |
| Aminopeptidase N | P15144 | 51 | 0 | 0 |
| 15-hydroxyprostaglandin dehydrogenase [NAD(+)] | P15428 | 58 | 121905 | 2102 |
| Cytochrome P450 11B1, mitochondrial | P15538 | 337 | 0 | 0 |
| NAD(P)H dehydrogenase [quinone] 1 | P15559 | 56 | 415264 | 7415 |
| Phosphorylase b kinase gamma catalytic chain, liver/testis isoform | P15735 | 58 | 16 | 0 |
| Arachidonate 15-lipoxygenase | P16050 | 60 | 66830 | 1114 |
| Ribosyldihydronicotinamide dehydrogenase [quinone] | P16083 | 119 | 61 | 1 |
| P-selectin | P16109 | 19 | 37 | 2 |
| Platelet-derived growth factor receptor alpha | P16234 | 391 | 375810 | 961 |
| Dipeptidase 1 | P16444 | 10 | 0 | 0 |
| Methylated-DNA--protein-cysteine methyltransferase | P16455 | 30 | 64921 | 2164 |
| Rod cGMP-specific 3',5'-cyclic phosphodiesterase subunit alpha | P16499 | 49 | 0 | 0 |
| E-selectin | P16581 | 106 | 383440 | 3617 |
| Tyrosine-protein kinase Fer | P16591 | 115 | 227221 | 1976 |
| UDP-glucuronosyltransferase 2B7 | P16662 | 15 | 11381 | 759 |
| Protein kinase C alpha type | P17252 | 376 | 556894 | 1481 |
| Integrin alpha-2 | P17301 | 16 | 329633 | 20602 |
| Chymotrypsinogen B | P17538 | 34 | 18 | 1 |
| cAMP-dependent protein kinase catalytic subunit alpha | P17612 | 299 | 438735 | 1467 |
| Calpain-2 catalytic subunit | P17655 | 136 | 369218 | 2715 |
| Tyrosine-protein phosphatase non-receptor type 2 | P17706 | 138 | 347121 | 2515 |
| S-adenosylmethionine decarboxylase proenzyme | P17707 | 11 | 3 | 0 |
| Tryptophan 5-hydroxylase 1 | P17752 | 45 | 217687 | 4837 |
| Neuronal acetylcholine receptor subunit beta-2 | P17787 | 418 | 128 | 0 |
| X-box-binding protein 1 | P17861 | 40 | 92845 | 2321 |
| Galectin-3 | P17931 | 16 | 2189 | 137 |
| Insulin-like growth factor-binding protein 3 | P17936 | 39 | 342744 | 8788 |
| Vascular endothelial growth factor receptor 1 | P17948 | 805 | 373758 | 464 |
| Tyrosine-protein phosphatase non-receptor type 1 | P18031 | 557 | 417486 | 750 |
| Arachidonate 12-lipoxygenase, 12S-type | P18054 | 25 | 184799 | 7392 |
| Integrin beta-5 | P18084 | 134 | 67 | 1 |
| Alpha-2B adrenergic receptor | P18089 | 189 | 213682 | 1131 |
| 3-oxo-5-alpha-steroid 4-dehydrogenase 1 | P18405 | 287 | 11 | 0 |
| Receptor-type tyrosine-protein phosphatase alpha | P18433 | 13 | 128 | 10 |
| Gamma-aminobutyric acid receptor subunit gamma-2 | P18507 | 811 | 418 | 1 |
| Integrin beta-6 | P18564 | 62 | 3 | 0 |
| Alpha-2C adrenergic receptor | P18825 | 321 | 216761 | 675 |
| Cytochrome P450 11B2, mitochondrial | P19099 | 411 | 4 | 0 |
| Vascular cell adhesion protein 1 | P19320 | 28 | 331382 | 11835 |
| Troponin I, cardiac muscle | P19429 | 17 | 332138 | 19538 |
| Tumor necrosis factor receptor superfamily member 1A | P19438 | 18 | 414633 | 23035 |
| Interferon-induced, double-stranded RNA-activated protein kinase | P19525 | 20 | 434102 | 21705 |
| Sodium/hydrogen exchanger 1 | P19634 | 140 | 175736 | 1255 |
| Casein kinase II subunit alpha' | P19784 | 48 | 422239 | 8797 |
| Retinoic acid receptor RXR-alpha | P19793 | 307 | 435869 | 1420 |
| Nuclear factor NF-kappa-B p105 subunit | P19838 | 171 | 425283 | 2487 |
| Thymidine phosphorylase | P19971 | 21 | 16 | 1 |
| Gastricsin | P20142 | 16 | 0 | 0 |
| Cyclin-A2 | P20248 | 526 | 415670 | 790 |
| Arachidonate 5-lipoxygenase-activating protein | P20292 | 233 | 34 | 0 |
| Muscarinic acetylcholine receptor M3 | P20309 | 806 | 101 | 0 |
| Nuclear receptor subfamily 1 group D member 1 | P20393 | 45 | 359381 | 7986 |
| Potassium-transporting ATPase alpha chain 1 | P20648 | 77 | 0 | 0 |
| Integrin alpha-L | P20701 | 339 | 27 | 0 |
| Inosine-5'-monophosphate dehydrogenase 1 | P20839 | 56 | 10 | 0 |
| Amine oxidase [flavin-containing] A | P21397 | 248 | 453 | 2 |
| Substance-K receptor | P21452 | 450 | 4 | 0 |
| Sphingosine 1-phosphate receptor 1 | P21453 | 838 | 382638 | 457 |
| fMet-Leu-Phe receptor | P21462 | 58 | 330472 | 5698 |
| Cannabinoid receptor 1 | P21554 | 2330 | 335422 | 144 |
| Ephrin type-A receptor 1 | P21709 | 12 | 0 | 0 |
| D(1A) dopamine receptor | P21728 | 442 | 357927 | 810 |
| C5a anaphylatoxin chemotactic receptor 1 | P21730 | 141 | 333462 | 2365 |
| Thromboxane A2 receptor | P21731 | 603 | 213658 | 354 |
| Fibroblast growth factor receptor 2 | P21802 | 89 | 362592 | 4074 |
| Ryanodine receptor 1 | P21817 | 10 | 67251 | 6725 |
| D(4) dopamine receptor | P21917 | 1336 | 16 | 0 |
| D(1B) dopamine receptor | P21918 | 113 | 380 | 3 |
| Catechol O-methyltransferase | P21964 | 11 | 2 | 0 |
| Protein-glutamine gamma-glutamyltransferase 2 | P21980 | 146 | 425046 | 2911 |
| Potassium voltage-gated channel subfamily A member 3 | P22001 | 190 | 7 | 0 |
| Trifunctional purine biosynthetic protein adenosine-3 | P22102 | 32 | 15 | 0 |
| Acetylcholinesterase | P22303 | 1060 | 193058 | 182 |
| Fibroblast growth factor receptor 4 | P22455 | 51 | 375839 | 7369 |
| Potassium voltage-gated channel subfamily A member 5 | P22460 | 333 | 102 | 0 |
| Fibroblast growth factor receptor 3 | P22607 | 162 | 202368 | 1249 |
| cAMP-dependent protein kinase catalytic subunit gamma | P22612 | 30 | 0 | 0 |
| cAMP-dependent protein kinase catalytic subunit beta | P22694 | 34 | 337578 | 9929 |
| Nuclear receptor subfamily 4 group A member 1 | P22736 | 11 | 471383 | 42853 |
| Carbonic anhydrase 4 | P22748 | 115 | 1 | 0 |
| Lutropin-choriogonadotropic hormone receptor | P22888 | 12 | 354459 | 29538 |
| Neutrophil collagenase | P22894 | 678 | 3 | 0 |
| Liver carboxylesterase 1 | P23141 | 139 | 44 | 0 |
| Prostaglandin G/H synthase 1 | P23219 | 258 | 45 | 0 |
| Carbonic anhydrase 6 | P23280 | 55 | 0 | 0 |
| Ribosomal protein S6 kinase beta-1 | P23443 | 274 | 415390 | 1516 |
| Tyrosine-protein kinase JAK1 | P23458 | 305 | 387201 | 1270 |
| Receptor-type tyrosine-protein phosphatase beta | P23467 | 35 | 38 | 1 |
| Adenosylhomocysteinase | P23526 | 48 | 5 | 0 |
| Ribonucleoside-diphosphate reductase large subunit | P23921 | 35 | 156879 | 4482 |
| Follicle-stimulating hormone receptor | P23945 | 33 | 323508 | 9803 |
| Chymase | P23946 | 252 | 30 | 0 |
| Sodium-dependent noradrenaline transporter | P23975 | 1634 | 26 | 0 |
| G1/S-specific cyclin-D1 | P24385 | 476 | 421271 | 885 |
| Endothelin B receptor | P24530 | 494 | 10 | 0 |
| Thromboxane-A synthase | P24557 | 570 | 3 | 0 |
| Protein kinase C eta type | P24723 | 162 | 195668 | 1208 |
| G1/S-specific cyclin-E1 | P24864 | 620 | 381691 | 616 |
| Cyclin-dependent kinase 2 | P24941 | 1979 | 422731 | 214 |
| Histamine H2 receptor | P25021 | 77 | 23 | 0 |
| C-X-C chemokine receptor type 1 | P25024 | 133 | 3968 | 30 |
| C-X-C chemokine receptor type 2 | P25025 | 389 | 348863 | 897 |
| N-formyl peptide receptor 2 | P25090 | 36 | 25602 | 711 |
| Beta-adrenergic receptor kinase 1 | P25098 | 12 | 373687 | 31141 |
| Alpha-1D adrenergic receptor | P25100 | 561 | 3 | 0 |
| Endothelin-1 receptor | P25101 | 1020 | 23 | 0 |
| Substance-P receptor | P25103 | 1763 | 25 | 0 |
| Platelet-activating factor receptor | P25105 | 295 | 381055 | 1292 |
| Atypical chemokine receptor 3 | P25106 | 16 | 1 | 0 |
| Proteinase-activated receptor 1 | P25116 | 407 | 307366 | 755 |
| Bromodomain-containing protein 2 | P25440 | 15 | 42 | 3 |
| Cathepsin S | P25774 | 1184 | 365972 | 309 |
| Neuropeptide Y receptor type 1 | P25929 | 362 | 215852 | 596 |
| Integrin beta-7 | P26010 | 330 | 325165 | 985 |
| Threonine--tRNA ligase, cytoplasmic | P26639 | 15 | 0 | 0 |
| Amine oxidase [flavin-containing] B | P27338 | 599 | 239 | 0 |
| Mitogen-activated protein kinase 3 | P27361 | 33 | 469332 | 14222 |
| MAP/microtubule affinity-regulating kinase 3 | P27448 | 132 | 304395 | 2306 |
| Dipeptidyl peptidase 4 | P27487 | 2410 | 64 | 0 |
| Deoxycytidine kinase | P27707 | 84 | 11 | 0 |
| cAMP-specific 3',5'-cyclic phosphodiesterase 4A | P27815 | 561 | 18 | 0 |
| Phosphatidylinositol 3-kinase regulatory subunit alpha | P27986 | 100 | 479599 | 4796 |
| Proteasome subunit beta type-8 | P28062 | 10 | 1 | 0 |
| Proteasome subunit beta type-5 | P28074 | 80 | 342118 | 4276 |
| 5-hydroxytryptamine receptor 1D | P28221 | 829 | 81108 | 98 |
| 5-hydroxytryptamine receptor 1B | P28222 | 729 | 64727 | 89 |
| 5-hydroxytryptamine receptor 2A | P28223 | 1685 | 325795 | 193 |
| 5-hydroxytryptamine receptor 2C | P28335 | 1469 | 124 | 0 |
| Neuromedin-B receptor | P28336 | 61 | 0 | 0 |
| Gamma-aminobutyric acid receptor subunit beta-3 | P28472 | 782 | 14 | 0 |
| Mitogen-activated protein kinase 1 | P28482 | 148 | 480015 | 3243 |
| Retinoic acid receptor RXR-beta | P28702 | 98 | 415966 | 4245 |
| DNA repair protein complementing XP-G cells | P28715 | 32 | 26 | 1 |
| Cytosol aminopeptidase | P28838 | 22 | 45 | 2 |
| Corticosteroid 11-beta-dehydrogenase isozyme 1 | P28845 | 1509 | 32 | 0 |
| Adenosine receptor A2a | P29274 | 2403 | 400917 | 167 |
| Adenosine receptor A2b | P29275 | 1267 | 34 | 0 |
| Ephrin type-A receptor 2 | P29317 | 106 | 358770 | 3385 |
| Ephrin type-A receptor 3 | P29320 | 12 | 429577 | 35798 |
| Ephrin type-B receptor 2 | P29323 | 11 | 359189 | 32654 |
| Neuromedin-K receptor | P29371 | 369 | 10 | 0 |
| Leukocyte tyrosine kinase receptor | P29376 | 106 | 151 | 1 |
| Transketolase | P29401 | 43 | 0 | 0 |
| Caspase-1 | P29466 | 380 | 432466 | 1138 |
| Nitric oxide synthase, endothelial | P29474 | 87 | 362803 | 4170 |
| Nitric oxide synthase, brain | P29475 | 334 | 341845 | 1023 |
| Non-receptor tyrosine-protein kinase TYK2 | P29597 | 243 | 194528 | 801 |
| G1/S-specific cyclin-D2 | P30279 | 109 | 343373 | 3150 |
| G1/S-specific cyclin-D3 | P30281 | 116 | 424374 | 3658 |
| Wee1-like protein kinase | P30291 | 240 | 488904 | 2037 |
| M-phase inducer phosphatase 1 | P30304 | 15 | 407041 | 27136 |
| M-phase inducer phosphatase 2 | P30305 | 47 | 225025 | 4788 |
| B2 bradykinin receptor | P30411 | 319 | 323475 | 1014 |
| Glycylpeptide N-tetradecanoyltransferase 1 | P30419 | 15 | 365098 | 24340 |
| Vasopressin V2 receptor | P30518 | 342 | 339620 | 993 |
| Tyrosine-protein kinase receptor UFO | P30530 | 218 | 362835 | 1664 |
| Sodium- and chloride-dependent GABA transporter 1 | P30531 | 39 | 5 | 0 |
| Translocator protein | P30536 | 12 | 1 | 0 |
| Adenosine receptor A1 | P30542 | 2055 | 387934 | 189 |
| Gastrin-releasing peptide receptor | P30550 | 17 | 0 | 0 |
| Type-1 angiotensin II receptor | P30556 | 363 | 372916 | 1027 |
| Oxytocin receptor | P30559 | 347 | 320139 | 923 |
| Somatostatin receptor type 1 | P30872 | 43 | 3 | 0 |
| Somatostatin receptor type 2 | P30874 | 148 | 223868 | 1513 |
| Neuronal acetylcholine receptor subunit beta-4 | P30926 | 125 | 174 | 1 |
| 5-hydroxytryptamine receptor 1F | P30939 | 91 | 0 | 0 |
| Gonadotropin-releasing hormone receptor | P30968 | 919 | 1 | 0 |
| Neurotensin receptor type 1 | P30989 | 72 | 327007 | 4542 |
| 3-oxo-5-alpha-steroid 4-dehydrogenase 2 | P31213 | 252 | 4 | 0 |
| Ribonucleoside-diphosphate reductase subunit M2 | P31350 | 38 | 365066 | 9607 |
| Somatostatin receptor type 4 | P31391 | 69 | 3 | 0 |
| Sodium/glucose cotransporter 2 | P31639 | 584 | 0 | 0 |
| Gamma-aminobutyric acid receptor subunit alpha-5 | P31644 | 576 | 0 | 0 |
| Sodium-dependent serotonin transporter | P31645 | 2651 | 12157 | 5 |
| RAC-alpha serine/threonine-protein kinase | P31749 | 924 | 512563 | 555 |
| RAC-beta serine/threonine-protein kinase | P31751 | 370 | 395928 | 1070 |
| Cholecystokinin receptor type A | P32238 | 163 | 7 | 0 |
| Gastrin/cholecystokinin type B receptor | P32239 | 540 | 60 | 0 |
| Melanocortin receptor 4 | P32245 | 1500 | 355916 | 237 |
| C-C chemokine receptor type 1 | P32246 | 452 | 202301 | 448 |
| Bombesin receptor subtype-3 | P32247 | 153 | 2 | 0 |
| Neuronal acetylcholine receptor subunit alpha-3 | P32297 | 156 | 103 | 1 |
| Somatostatin receptor type 3 | P32745 | 114 | 374745 | 3287 |
| Melanocortin receptor 5 | P33032 | 179 | 22 | 0 |
| Cytochrome P450 2C19 | P33261 | 15 | 101326 | 6755 |
| Deoxyuridine 5'-triphosphate nucleotidohydrolase, mitochondrial | P33316 | 40 | 93215 | 2330 |
| Multidrug resistance-associated protein 1 | P33527 | 86 | 140631 | 1635 |
| Adenosine receptor A3 | P33765 | 2277 | 40 | 0 |
| Dual specificity protein kinase TTK | P33981 | 82 | 192540 | 2348 |
| Gamma-aminobutyric acid receptor subunit alpha-3 | P34903 | 583 | 0 | 0 |
| Bifunctional epoxide hydrolase 2 | P34913 | 1031 | 90204 | 87 |
| G protein-coupled receptor kinase 5 | P34947 | 38 | 367388 | 9668 |
| 5-hydroxytryptamine receptor 7 | P34969 | 699 | 17 | 0 |
| Cannabinoid receptor 2 | P34972 | 2638 | 3679 | 1 |
| Prostaglandin E2 receptor EP1 subtype | P34995 | 174 | 1239 | 7 |
| Corticotropin-releasing factor receptor 1 | P34998 | 1281 | 353797 | 276 |
| Trypsin-3 | P35030 | 101 | 192904 | 1910 |
| Carbonic anhydrase 5A, mitochondrial | P35218 | 94 | 0 | 0 |
| Nitric oxide synthase, inducible | P35228 | 375 | 192883 | 514 |
| Tyrosine-protein phosphatase non-receptor type 7 | P35236 | 79 | 230756 | 2921 |
| Somatostatin receptor type 5 | P35346 | 148 | 401358 | 2712 |
| Alpha-1A adrenergic receptor | P35348 | 822 | 53 | 0 |
| Prostaglandin G/H synthase 2 | P35354 | 1303 | 365114 | 280 |
| Histamine H1 receptor | P35367 | 692 | 25 | 0 |
| Alpha-1B adrenergic receptor | P35368 | 637 | 338572 | 532 |
| Mu-type opioid receptor | P35372 | 2027 | 349474 | 172 |
| Prostaglandin E2 receptor EP4 subtype | P35408 | 247 | 9 | 0 |
| D(3) dopamine receptor | P35462 | 1800 | 411916 | 229 |
| Sodium channel protein type 4 subunit alpha | P35499 | 13 | 0 | 0 |
| Glucokinase | P35557 | 316 | 128899 | 408 |
| Phosphoenolpyruvate carboxykinase, cytosolic [GTP] | P35558 | 11 | 0 | 0 |
| Sterol O-acyltransferase 1 | P35610 | 115 | 4 | 0 |
| Aryl hydrocarbon receptor | P35869 | 47 | 389515 | 8288 |
| Vascular endothelial growth factor receptor 3 | P35916 | 448 | 329791 | 736 |
| Vascular endothelial growth factor receptor 2 | P35968 | 3334 | 374684 | 112 |
| Dual specificity mitogen-activated protein kinase kinase 2 | P36507 | 66 | 342114 | 5184 |
| Neuronal acetylcholine receptor subunit alpha-7 | P36544 | 275 | 192924 | 702 |
| Receptor-type tyrosine-protein kinase FLT3 | P36888 | 968 | 80 | 0 |
| Activin receptor type-1B | P36896 | 10 | 2 | 0 |
| TGF-beta receptor type-1 | P36897 | 441 | 410601 | 931 |
| Serine/threonine-protein kinase receptor R3 | P37023 | 12 | 193913 | 16159 |
| Testosterone 17-beta-dehydrogenase 3 | P37058 | 144 | 27 | 0 |
| Estradiol 17-beta-dehydrogenase 2 | P37059 | 200 | 69 | 0 |
| Amiloride-sensitive sodium channel subunit alpha | P37088 | 36 | 287376 | 7983 |
| TGF-beta receptor type-2 | P37173 | 38 | 403094 | 10608 |
| Peroxisome proliferator-activated receptor gamma | P37231 | 1460 | 428055 | 293 |
| Squalene synthase | P37268 | 140 | 22 | 0 |
| Vasopressin V1a receptor | P37288 | 456 | 319557 | 701 |
| Glutamate receptor ionotropic, kainate 1 | P39086 | 67 | 7 | 0 |
| Flap endonuclease 1 | P39748 | 30 | 414156 | 13805 |
| Calcium-dependent phospholipase A2 | P39877 | 15 | 15 | 1 |
| Macrophage metalloelastase | P39900 | 241 | 217721 | 903 |
| Thrombopoietin receptor | P40238 | 179 | 214749 | 1200 |
| Signal transducer and activator of transcription 3 | P40763 | 92 | 433862 | 4716 |
| Delta-type opioid receptor | P41143 | 1873 | 364122 | 194 |
| Kappa-type opioid receptor | P41145 | 1949 | 353461 | 181 |
| Nociceptin receptor | P41146 | 935 | 19419 | 21 |
| Extracellular calcium-sensing receptor | P41180 | 329 | 52 | 0 |
| P2Y purinoceptor 2 | P41231 | 88 | 180 | 2 |
| Tyrosine-protein kinase CSK | P41240 | 14 | 342151 | 24439 |
| Isoleucine--tRNA ligase, cytoplasmic | P41252 | 15 | 6640 | 443 |
| Mitogen-activated protein kinase kinase kinase 8 | P41279 | 129 | 396633 | 3075 |
| Pituitary adenylate cyclase-activating polypeptide type I receptor | P41586 | 15 | 8 | 1 |
| Metabotropic glutamate receptor 5 | P41594 | 991 | 223886 | 226 |
| 5-hydroxytryptamine receptor 2B | P41595 | 632 | 125 | 0 |
| C-C chemokine receptor type 2 | P41597 | 998 | 214257 | 215 |
| Protein kinase C iota type | P41743 | 22 | 312992 | 14227 |
| Melanocortin receptor 3 | P41968 | 251 | 28 | 0 |
| Signal transducer and activator of transcription 1-alpha/beta | P42224 | 30 | 472910 | 15764 |
| Signal transducer and activator of transcription 6 | P42226 | 60 | 366155 | 6103 |
| Glutamate receptor 1 | P42261 | 40 | 405346 | 10134 |
| Glutamate receptor 2 | P42262 | 76 | 400958 | 5276 |
| Aldo-keto reductase family 1 member C3 | P42330 | 188 | 34 | 0 |
| Phosphatidylinositol 4,5-bisphosphate 3-kinase catalytic subunit alpha isoform | P42336 | 1533 | 189557 | 124 |
| Phosphatidylinositol 4,5-bisphosphate 3-kinase catalytic subunit beta isoform | P42338 | 582 | 36 | 0 |
| Serine/threonine-protein kinase mTOR | P42345 | 953 | 407966 | 428 |
| Caspase-3 | P42574 | 623 | 447684 | 719 |
| Caspase-2 | P42575 | 26 | 382005 | 14693 |
| Megakaryocyte-associated tyrosine-protein kinase | P42679 | 30 | 64552 | 2152 |
| Tyrosine-protein kinase TXK | P42681 | 25 | 119 | 5 |
| Abelson tyrosine-protein kinase 2 | P42684 | 24 | 352746 | 14698 |
| Tyrosine-protein kinase FRK | P42685 | 158 | 10 | 0 |
| Lysosomal Pro-X carboxypeptidase | P42785 | 230 | 1 | 0 |
| Endothelin-converting enzyme 1 | P42892 | 197 | 1 | 0 |
| Excitatory amino acid transporter 1 | P43003 | 16 | 18 | 1 |
| Excitatory amino acid transporter 2 | P43004 | 28 | 19 | 1 |
| Excitatory amino acid transporter 3 | P43005 | 18 | 61 | 3 |
| Prostaglandin F2-alpha receptor | P43088 | 41 | 255456 | 6231 |
| Prostaglandin E2 receptor EP3 subtype | P43115 | 435 | 1200 | 3 |
| Prostaglandin E2 receptor EP2 subtype | P43116 | 48 | 255485 | 5323 |
| Prostacyclin receptor | P43119 | 89 | 339629 | 3816 |
| Carbonic anhydrase 7 | P43166 | 286 | 0 | 0 |
| Glucagon-like peptide 1 receptor | P43220 | 16 | 400027 | 25002 |
| Cathepsin K | P43235 | 1177 | 22 | 0 |
| Nuclear receptor subfamily 4 group A member 2 | P43354 | 10 | 357121 | 35712 |
| Tyrosine-protein kinase ZAP-70 | P43403 | 77 | 414594 | 5384 |
| Tyrosine-protein kinase SYK | P43405 | 325 | 439061 | 1351 |
| Nicotinamide phosphoribosyltransferase | P43490 | 197 | 0 | 0 |
| Neuronal acetylcholine receptor subunit alpha-4 | P43681 | 457 | 125 | 0 |
| Troponin T, cardiac muscle | P45379 | 17 | 330272 | 19428 |
| Collagenase 3 | P45452 | 1776 | 362437 | 204 |
| Mitogen-activated protein kinase 8 | P45983 | 679 | 433138 | 638 |
| Mitogen-activated protein kinase 9 | P45984 | 485 | 430812 | 888 |
| Dual specificity mitogen-activated protein kinase kinase 4 | P45985 | 12 | 354033 | 29503 |
| 5-hydroxytryptamine receptor 3A | P46098 | 349 | 402953 | 1155 |
| B1 bradykinin receptor | P46663 | 600 | 7 | 0 |
| Cytosolic phospholipase A2 | P47712 | 133 | 332253 | 2498 |
| Gamma-aminobutyric acid receptor subunit alpha-2 | P47869 | 494 | 0 | 0 |
| Gamma-aminobutyric acid receptor subunit beta-2 | P47870 | 69 | 18 | 0 |
| Glucagon receptor | P47871 | 509 | 339609 | 667 |
| 5-hydroxytryptamine receptor 5A | P47898 | 81 | 330277 | 4077 |
| P2Y purinoceptor 1 | P47900 | 175 | 111 | 1 |
| Vasopressin V1b receptor | P47901 | 183 | 226 | 1 |
| Xanthine dehydrogenase/oxidase | P47989 | 19 | 0 | 0 |
| Melatonin receptor type 1A | P48039 | 551 | 435724 | 791 |
| ATP-sensitive inward rectifier potassium channel 1 | P48048 | 42 | 123385 | 2938 |
| G protein-activated inward rectifier potassium channel 2 | P48051 | 21 | 401904 | 19138 |
| Glutamate receptor 4 | P48058 | 64 | 64789 | 1012 |
| Sodium- and chloride-dependent glycine transporter 1 | P48067 | 430 | 48 | 0 |
| Neuropeptides B/W receptor type 1 | P48145 | 85 | 286311 | 3368 |
| Prolyl endopeptidase | P48147 | 109 | 24 | 0 |
| Gamma-aminobutyric acid receptor subunit alpha-4 | P48169 | 18 | 21 | 1 |
| Retinoic acid receptor RXR-gamma | P48443 | 96 | 432337 | 4504 |
| Lanosterol synthase | P48449 | 82 | 0 | 0 |
| G protein-activated inward rectifier potassium channel 4 | P48544 | 24 | 1349 | 56 |
| Gastric inhibitory polypeptide receptor | P48546 | 73 | 1 | 0 |
| G protein-activated inward rectifier potassium channel 1 | P48549 | 30 | 1349 | 45 |
| Casein kinase I isoform alpha | P48729 | 156 | 398363 | 2554 |
| Casein kinase I isoform delta | P48730 | 190 | 421236 | 2217 |
| Phosphatidylinositol 4,5-bisphosphate 3-kinase catalytic subunit gamma isoform | P48736 | 702 | 341829 | 487 |
| Hydroxycarboxylic acid receptor 3 | P49019 | 16 | 82 | 5 |
| MAP kinase-activated protein kinase 2 | P49137 | 471 | 362473 | 770 |
| Neuropeptide Y receptor type 2 | P49146 | 125 | 230254 | 1842 |
| CX3C chemokine receptor 1 | P49238 | 43 | 1 | 0 |
| Natural resistance-associated macrophage protein 2 | P49281 | 30 | 0 | 0 |
| Melatonin receptor type 1B | P49286 | 515 | 435368 | 845 |
| Fatty acid synthase | P49327 | 52 | 354352 | 6814 |
| Cyclin-dependent kinase 8 | P49336 | 133 | 15 | 0 |
| Protein farnesyltransferase/geranylgeranyltransferase type-1 subunit alpha | P49354 | 1113 | 193251 | 174 |
| Protein farnesyltransferase subunit beta | P49356 | 1074 | 193248 | 180 |
| Casein kinase I isoform epsilon | P49674 | 26 | 75 | 3 |
| C-X-C chemokine receptor type 3 | P49682 | 555 | 1 | 0 |
| Proteasome subunit beta type-2 | P49721 | 14 | 342131 | 24438 |
| Dual specificity protein kinase CLK1 | P49759 | 100 | 157031 | 1570 |
| Dual specificity protein kinase CLK2 | P49760 | 285 | 157044 | 551 |
| Dual specificity protein kinase CLK3 | P49761 | 12 | 156941 | 13078 |
| Presenilin-1 | P49768 | 190 | 443645 | 2335 |
| Regulator of G-protein signaling 4 | P49798 | 39 | 389756 | 9994 |
| Presenilin-2 | P49810 | 188 | 401486 | 2136 |
| Glycogen synthase kinase-3 alpha | P49840 | 453 | 491849 | 1086 |
| Glycogen synthase kinase-3 beta | P49841 | 1520 | 447479 | 294 |
| Type-2 angiotensin II receptor | P50052 | 286 | 7 | 0 |
| Ketohexokinase | P50053 | 75 | 0 | 0 |
| Matrix metalloproteinase-14 | P50281 | 306 | 362473 | 1185 |
| 5-hydroxytryptamine receptor 6 | P50406 | 1639 | 339602 | 207 |
| Methionine aminopeptidase 2 | P50579 | 335 | 386208 | 1153 |
| Cyclin-dependent kinase 7 | P50613 | 213 | 408162 | 1916 |
| Cyclin-dependent kinase 9 | P50750 | 254 | 464051 | 1827 |
| Cone cGMP-specific 3',5'-cyclic phosphodiesterase subunit alpha' | P51160 | 15 | 4 | 0 |
| Potassium-transporting ATPase subunit beta | P51164 | 77 | 0 | 0 |
| Tyrosine-protein kinase Blk | P51451 | 211 | 192640 | 913 |
| Dual specificity protein phosphatase 3 | P51452 | 64 | 341114 | 5330 |
| Matrix metalloproteinase-15 | P51511 | 12 | 7 | 1 |
| Matrix metalloproteinase-16 | P51512 | 30 | 7 | 0 |
| P2Y purinoceptor 4 | P51582 | 28 | 32 | 1 |
| Interleukin-1 receptor-associated kinase 1 | P51617 | 94 | 383910 | 4084 |
| C-C chemokine receptor type 3 | P51677 | 835 | 25 | 0 |
| C-C chemokine receptor type 4 | P51679 | 176 | 323262 | 1837 |
| C-C chemokine receptor type 5 | P51681 | 1514 | 356259 | 235 |
| C-C chemokine receptor type 8 | P51685 | 125 | 2 | 0 |
| Potassium voltage-gated channel subfamily KQT member 1 | P51787 | 17 | 302337 | 17785 |
| Ribosomal protein S6 kinase alpha-3 | P51812 | 270 | 319037 | 1182 |
| Cytoplasmic tyrosine-protein kinase BMX | P51813 | 13 | 333194 | 25630 |
| cAMP-dependent protein kinase catalytic subunit PRKX | P51817 | 198 | 0 | 0 |
| Cyclin-H | P51946 | 52 | 368984 | 7096 |
| Serine/threonine-protein kinase Nek2 | P51955 | 123 | 323724 | 2632 |
| Serine/threonine-protein kinase Nek4 | P51957 | 67 | 0 | 0 |
| Tyrosine-protein kinase JAK3 | P52333 | 585 | 223663 | 382 |
| Kinesin-like protein KIF11 | P52732 | 486 | 59 | 0 |
| Aldo-keto reductase family 1 member C2 | P52895 | 34 | 6 | 0 |
| Serine/threonine-protein kinase PLK1 | P53350 | 373 | 422683 | 1133 |
| ATP-citrate synthase | P53396 | 12 | 302773 | 25231 |
| Methionine aminopeptidase 1 | P53582 | 62 | 22 | 0 |
| Geranylgeranyl transferase type-1 subunit beta | P53609 | 208 | 193198 | 929 |
| Dipeptidyl peptidase 1 | P53634 | 52 | 300441 | 5778 |
| LIM domain kinase 1 | P53667 | 155 | 213667 | 1378 |
| LIM domain kinase 2 | P53671 | 59 | 27 | 0 |
| Mitogen-activated protein kinase 12 | P53778 | 167 | 266410 | 1595 |
| Mitogen-activated protein kinase 10 | P53779 | 456 | 409747 | 899 |
| Monocarboxylate transporter 1 | P53985 | 43 | 0 | 0 |
| Voltage-dependent calcium channel subunit alpha-2/delta-1 | P54289 | 123 | 0 | 0 |
| Tyrosine--tRNA ligase, cytoplasmic | P54577 | 12 | 74 | 6 |
| 5'-AMP-activated protein kinase catalytic subunit alpha-2 | P54646 | 10 | 566 | 57 |
| Calcium/calmodulin-dependent 3',5'-cyclic nucleotide phosphodiesterase 1A | P54750 | 12 | 8 | 1 |
| Ephrin type-B receptor 3 | P54753 | 19 | 60 | 3 |
| Ephrin type-B receptor 4 | P54760 | 207 | 96394 | 466 |
| Ephrin type-B receptor 1 | P54762 | 10 | 356681 | 35668 |
| Ephrin type-A receptor 4 | P54764 | 10 | 28 | 3 |
| Oxysterols receptor LXR-beta | P55055 | 452 | 409788 | 907 |
| Transitional endoplasmic reticulum ATPase | P55072 | 74 | 398168 | 5381 |
| Proteinase-activated receptor 2 | P55085 | 30 | 362542 | 12085 |
| Microsomal triglyceride transfer protein large subunit | P55157 | 148 | 0 | 0 |
| Caspase-7 | P55210 | 228 | 432733 | 1898 |
| Caspase-9 | P55211 | 10 | 372538 | 37254 |
| Caspase-6 | P55212 | 60 | 437559 | 7293 |
| Adenosine kinase | P55263 | 302 | 10 | 0 |
| Methionine--tRNA ligase, cytoplasmic | P56192 | 28 | 0 | 0 |
| P2X purinoceptor 3 | P56373 | 41 | 6 | 0 |
| Histone deacetylase 4 | P56524 | 353 | 454619 | 1288 |
| Proto-oncogene Wnt-3 | P56703 | 37 | 10 | 0 |
| Protein Wnt-3a | P56704 | 15 | 17 | 1 |
| Beta-secretase 1 | P56817 | 1839 | 219571 | 119 |
| Serine/threonine-protein kinase SIK1 | P57059 | 13 | 213656 | 16435 |
| Eukaryotic initiation factor 4A-I | P60842 | 25 | 214871 | 8595 |
| C-X-C chemokine receptor type 4 | P61073 | 136 | 238166 | 1751 |
| Ubiquitin-conjugating enzyme E2 N | P61088 | 13 | 326665 | 25128 |
| WD repeat-containing protein 5 | P61964 | 12 | 299220 | 24935 |
| Estrogen-related receptor gamma | P62508 | 13 | 357419 | 27494 |
| Peptidyl-prolyl cis-trans isomerase A | P62937 | 48 | 214251 | 4464 |
| Peptidyl-prolyl cis-trans isomerase FKBP1A | P62942 | 265 | 47005 | 177 |
| Growth factor receptor-bound protein 2 | P62993 | 91 | 442574 | 4863 |
| Troponin C, slow skeletal and cardiac muscles | P63316 | 17 | 330245 | 19426 |
| Casein kinase II subunit beta | P67870 | 25 | 435392 | 17416 |
| Tubulin alpha-1B chain | P68363 | 25 | 332868 | 13315 |
| Tubulin alpha-4A chain | P68366 | 25 | 504231 | 20169 |
| Tubulin beta-4B chain | P68371 | 25 | 5 | 0 |
| Casein kinase II subunit alpha | P68400 | 217 | 447853 | 2064 |
| Casein kinase I isoform gamma-2 | P78368 | 106 | 17 | 0 |
| Cyclin-A1 | P78396 | 507 | 355978 | 702 |
| DNA-dependent protein kinase catalytic subunit | P78527 | 378 | 502152 | 1328 |
| Disintegrin and metalloproteinase domain-containing protein 17 | P78536 | 1253 | 375573 | 300 |
| HLA class II histocompatibility antigen, DR beta 3 chain | P79483 | 11 | 0 | 0 |
| Mitogen-activated protein kinase kinase kinase 9 | P80192 | 42 | 0 | 0 |
| Corticosteroid 11-beta-dehydrogenase isozyme 2 | P80365 | 43 | 94 | 2 |
| E3 ubiquitin-protein ligase XIAP | P98170 | 244 | 383500 | 1572 |
| Cyclin-dependent kinase 6 | Q00534 | 18 | 229487 | 12749 |
| Cyclin-dependent kinase 5 | Q00535 | 803 | 419172 | 522 |
| Cyclin-dependent kinase 16 | Q00536 | 14 | 421344 | 30096 |
| Cyclin-dependent kinase 17 | Q00537 | 12 | 304401 | 25367 |
| Nuclear factor NF-kappa-B p100 subunit | Q00653 | 162 | 374850 | 2314 |
| Sorbitol dehydrogenase | Q00796 | 55 | 16 | 0 |
| Voltage-dependent N-type calcium channel subunit alpha-1B | Q00975 | 155 | 299988 | 1935 |
| E3 ubiquitin-protein ligase Mdm2 | Q00987 | 216 | 432594 | 2003 |
| Runt-related transcription factor 1 | Q01196 | 10 | 231461 | 23146 |
| AMP deaminase 3 | Q01432 | 25 | 0 | 0 |
| Melanocyte-stimulating hormone receptor | Q01726 | 222 | 350078 | 1577 |
| Sodium-dependent dopamine transporter | Q01959 | 1232 | 405233 | 329 |
| Dihydroorotate dehydrogenase (quinone), mitochondrial | Q02127 | 222 | 0 | 0 |
| Protein kinase C epsilon type | Q02156 | 162 | 396505 | 2448 |
| Amyloid beta A4 precursor protein-binding family A member 1 | Q02410 | 11 | 303898 | 27627 |
| Dual specificity mitogen-activated protein kinase kinase 1 | Q02750 | 421 | 353247 | 839 |
| Angiopoietin-1 receptor | Q02763 | 435 | 57 | 0 |
| Mitogen-activated protein kinase kinase kinase 10 | Q02779 | 22 | 380943 | 17316 |
| Peroxisome proliferator-activated receptor delta | Q03181 | 801 | 314192 | 392 |
| Transcription factor p65 | Q04206 | 177 | 438654 | 2478 |
| Glutamate carboxypeptidase 2 | Q04609 | 123 | 2 | 0 |
| Protein kinase C theta type | Q04759 | 465 | 473037 | 1017 |
| Lactoylglutathione lyase | Q04760 | 18 | 0 | 0 |
| Activin receptor type-1 | Q04771 | 80 | 394296 | 4929 |
| Aldo-keto reductase family 1 member C1 | Q04828 | 18 | 7 | 0 |
| Macrophage-stimulating protein receptor | Q04912 | 121 | 374001 | 3091 |
| Tyrosine-protein phosphatase non-receptor type 12 | Q05209 | 10 | 359140 | 35914 |
| Focal adhesion kinase 1 | Q05397 | 251 | 444191 | 1770 |
| Hormone-sensitive lipase | Q05469 | 133 | 186171 | 1400 |
| Protein kinase C zeta type | Q05513 | 45 | 391548 | 8701 |
| Glutamate receptor ionotropic, NMDA 1 | Q05586 | 194 | 456029 | 2351 |
| Protein kinase C delta type | Q05655 | 513 | 504295 | 983 |
| Folylpolyglutamate synthase, mitochondrial | Q05932 | 13 | 1 | 0 |
| Tyrosine-protein phosphatase non-receptor type 11 | Q06124 | 33 | 420116 | 12731 |
| Tyrosine-protein kinase BTK | Q06187 | 163 | 443385 | 2720 |
| Tyrosine-protein kinase receptor TYRO3 | Q06418 | 178 | 894 | 5 |
| Acetylcholine receptor subunit delta | Q07001 | 13 | 99 | 8 |
| Glutamyl aminopeptidase | Q07075 | 23 | 0 | 0 |
| cAMP-specific 3',5'-cyclic phosphodiesterase 4B | Q07343 | 699 | 70906 | 101 |
| KH domain-containing, RNA-binding, signal transduction-associated protein 1 | Q07666 | 35 | 385662 | 11019 |
| Bcl-2-like protein 1 | Q07817 | 213 | 473690 | 2224 |
| Induced myeloid leukemia cell differentiation protein Mcl-1 | Q07820 | 136 | 355173 | 2612 |
| Peroxisome proliferator-activated receptor alpha | Q07869 | 1107 | 369132 | 333 |
| Activated CDC42 kinase 1 | Q07912 | 188 | 1484 | 8 |
| 1,25-dihydroxyvitamin D(3) 24-hydroxylase, mitochondrial | Q07973 | 10 | 0 | 0 |
| Epithelial discoidin domain-containing receptor 1 | Q08345 | 48 | 362523 | 7553 |
| cAMP-specific 3',5'-cyclic phosphodiesterase 4C | Q08493 | 243 | 1 | 0 |
| cAMP-specific 3',5'-cyclic phosphodiesterase 4D | Q08499 | 445 | 64747 | 145 |
| Adenylate cyclase type 1 | Q08828 | 43 | 0 | 0 |
| Tyrosine-protein kinase ITK/TSK | Q08881 | 514 | 362591 | 705 |
| ATP-binding cassette sub-family C member 8 | Q09428 | 21 | 2 | 0 |
| Alpha-1,6-mannosyl-glycoprotein 2-beta-N-acetylglucosaminyltransferase | Q10469 | 15 | 0 | 0 |
| Calcium-activated potassium channel subunit alpha-1 | Q12791 | 13 | 110661 | 8512 |
| Potassium voltage-gated channel subfamily H member 2 | Q12809 | 1166 | 308032 | 264 |
| Mitogen-activated protein kinase kinase kinase kinase 2 | Q12851 | 252 | 286221 | 1136 |
| Tyrosine-protein kinase Mer | Q12866 | 50 | 19 | 0 |
| Glutamate receptor ionotropic, NMDA 2A | Q12879 | 32 | 368465 | 11515 |
| Seprase | Q12884 | 26 | 26 | 1 |
| Ileal sodium/bile acid cotransporter | Q12908 | 154 | 0 | 0 |
| Glutamate receptor ionotropic, kainate 2 | Q13002 | 35 | 216743 | 6193 |
| Acetyl-CoA carboxylase 1 | Q13085 | 121 | 355890 | 2941 |
| Platelet-activating factor acetylhydrolase | Q13093 | 244 | 317237 | 1300 |
| S-methyl-5'-thioadenosine phosphorylase | Q13126 | 57 | 1 | 0 |
| 5'-AMP-activated protein kinase catalytic subunit alpha-1 | Q13131 | 168 | 417021 | 2482 |
| Oxysterols receptor LXR-alpha | Q13133 | 404 | 358405 | 887 |
| Serine/threonine-protein kinase PAK 1 | Q13153 | 41 | 468768 | 11433 |
| Dual specificity mitogen-activated protein kinase kinase 5 | Q13163 | 16 | 286052 | 17878 |
| Mitogen-activated protein kinase 7 | Q13164 | 23 | 10885 | 473 |
| Serine/threonine-protein kinase 3 | Q13188 | 207 | 322369 | 1557 |
| Glutamate receptor ionotropic, NMDA 2B | Q13224 | 205 | 417208 | 2035 |
| cGMP-dependent protein kinase 2 | Q13237 | 75 | 362742 | 4837 |
| Metabotropic glutamate receptor 1 | Q13255 | 377 | 339669 | 901 |
| Prostaglandin D2 receptor | Q13258 | 334 | 339604 | 1017 |
| Steroidogenic factor 1 | Q13285 | 51 | 384429 | 7538 |
| cGMP-inhibited 3',5'-cyclic phosphodiesterase B | Q13370 | 232 | 354599 | 1528 |
| Phospholipase D1 | Q13393 | 67 | 365874 | 5461 |
| Disintegrin and metalloproteinase domain-containing protein 9 | Q13443 | 20 | 2301 | 115 |
| Rho-associated protein kinase 1 | Q13464 | 522 | 324339 | 621 |
| Non-receptor tyrosine-protein kinase TNK1 | Q13470 | 10 | 362522 | 36252 |
| Baculoviral IAP repeat-containing protein 3 | Q13489 | 29 | 370482 | 12775 |
| Baculoviral IAP repeat-containing protein 2 | Q13490 | 61 | 332091 | 5444 |
| Short transient receptor potential channel 3 | Q13507 | 13 | 364766 | 28059 |
| Tubulin beta-3 chain | Q13509 | 30 | 53 | 2 |
| Peptidyl-prolyl cis-trans isomerase NIMA-interacting 1 | Q13526 | 31 | 425976 | 13741 |
| Serine/threonine-protein kinase ATR | Q13535 | 67 | 415542 | 6202 |
| Histone deacetylase 1 | Q13547 | 1543 | 452752 | 293 |
| Calcium/calmodulin-dependent protein kinase type II subunit beta | Q13554 | 29 | 422766 | 14578 |
| Calcium/calmodulin-dependent protein kinase type II subunit gamma | Q13555 | 65 | 417293 | 6420 |
| Calcium/calmodulin-dependent protein kinase type II subunit delta | Q13557 | 143 | 194355 | 1359 |
| Polycystin-2 | Q13563 | 28 | 330270 | 11795 |
| Dual specificity tyrosine-phosphorylation-regulated kinase 1A | Q13627 | 412 | 156938 | 381 |
| 5-hydroxytryptamine receptor 4 | Q13639 | 267 | 340596 | 1276 |
| Voltage-dependent L-type calcium channel subunit alpha-1S | Q13698 | 27 | 4 | 0 |
| Tubulin alpha-3C/D chain | Q13748 | 25 | 296885 | 11875 |
| Ectonucleotide pyrophosphatase/phosphodiesterase family member 2 | Q13822 | 34 | 80 | 2 |
| Bone morphogenetic protein receptor type-2 | Q13873 | 11 | 193474 | 17589 |
| Protein-tyrosine kinase 6 | Q13882 | 108 | 120 | 1 |
| Tubulin beta-2A chain | Q13885 | 25 | 5 | 0 |
| Voltage-dependent L-type calcium channel subunit alpha-1C | Q13936 | 42 | 64754 | 1542 |
| High affinity cAMP-specific 3',5'-cyclic phosphodiesterase 7A | Q13946 | 196 | 1 | 0 |
| Core-binding factor subunit beta | Q13951 | 10 | 225212 | 22521 |
| cGMP-dependent protein kinase 1 | Q13976 | 109 | 0 | 0 |
| Calcium/calmodulin-dependent protein kinase type 1 | Q14012 | 18 | 282119 | 15673 |
| Calcium/calmodulin-dependent 3',5'-cyclic nucleotide phosphodiesterase 1C | Q14123 | 31 | 0 | 0 |
| Inhibitor of nuclear factor kappa-B kinase subunit epsilon | Q14164 | 81 | 319141 | 3940 |
| Protein-tyrosine kinase 2-beta | Q14289 | 118 | 378362 | 3206 |
| Metabotropic glutamate receptor 2 | Q14416 | 322 | 225493 | 700 |
| cGMP-inhibited 3',5'-cyclic phosphodiesterase A | Q14432 | 275 | 38 | 0 |
| Sodium channel protein type 5 subunit alpha | Q14524 | 58 | 24 | 0 |
| Squalene monooxygenase | Q14534 | 21 | 0 | 0 |
| ATP-sensitive inward rectifier potassium channel 11 | Q14654 | 63 | 333502 | 5294 |
| Maternal embryonic leucine zipper kinase | Q14680 | 162 | 65036 | 401 |
| Membrane-bound transcription factor site-1 protease | Q14703 | 14 | 0 | 0 |
| Caspase-8 | Q14790 | 116 | 440633 | 3799 |
| Metabotropic glutamate receptor 3 | Q14832 | 40 | 19 | 0 |
| Metabotropic glutamate receptor 4 | Q14833 | 92 | 5287 | 57 |
| Lysine--tRNA ligase | Q15046 | 74 | 20 | 0 |
| Bromodomain-containing protein 3 | Q15059 | 29 | 0 | 0 |
| P2Y purinoceptor 6 | Q15077 | 40 | 23 | 1 |
| Cyclin-dependent kinase 5 activator 1 | Q15078 | 521 | 337595 | 648 |
| 3-beta-hydroxysteroid-Delta(8),Delta(7)-isomerase | Q15125 | 28 | 13 | 0 |
| Serine/threonine-protein kinase D1 | Q15139 | 102 | 383719 | 3762 |
| Receptor tyrosine-protein kinase erbB-4 | Q15303 | 86 | 368020 | 4279 |
| Ribosomal protein S6 kinase alpha-2 | Q15349 | 11 | 175889 | 15990 |
| P2Y purinoceptor 14 | Q15391 | 34 | 3 | 0 |
| Ribosomal protein S6 kinase alpha-1 | Q15418 | 36 | 445870 | 12385 |
| Tryptase alpha/beta-1 | Q15661 | 188 | 3 | 0 |
| Leukotriene B4 receptor 1 | Q15722 | 301 | 3497 | 12 |
| Myosin light chain kinase, smooth muscle | Q15746 | 28 | 90922 | 3247 |
| TGF-beta-activated kinase 1 and MAP3K7-binding protein 1 | Q15750 | 38 | 283283 | 7455 |
| Mitogen-activated protein kinase 11 | Q15759 | 220 | 322094 | 1464 |
| Neuropeptide Y receptor type 5 | Q15761 | 972 | 0 | 0 |
| Sodium channel protein type 9 subunit alpha | Q15858 | 1145 | 0 | 0 |
| NT-3 growth factor receptor | Q16288 | 192 | 376906 | 1963 |
| Gamma-aminobutyric acid receptor subunit alpha-6 | Q16445 | 58 | 0 | 0 |
| Glutamate receptor ionotropic, kainate 5 | Q16478 | 11 | 8 | 1 |
| Serine/threonine-protein kinase N2 | Q16513 | 179 | 372403 | 2080 |
| Mitogen-activated protein kinase 14 | Q16539 | 2669 | 470548 | 176 |
| Bcl-2-related protein A1 | Q16548 | 19 | 340151 | 17903 |
| Vesicular acetylcholine transporter | Q16572 | 152 | 0 | 0 |
| C3a anaphylatoxin chemotactic receptor | Q16581 | 18 | 24 | 1 |
| Mitogen-activated protein kinase kinase kinase 11 | Q16584 | 48 | 391539 | 8157 |
| Calcitonin gene-related peptide type 1 receptor | Q16602 | 477 | 1 | 0 |
| BDNF/NT-3 growth factors receptor | Q16620 | 173 | 362646 | 2096 |
| MAP kinase-activated protein kinase 3 | Q16644 | 10 | 5119 | 512 |
| Prostasin | Q16651 | 16 | 0 | 0 |
| Hypoxia-inducible factor 1-alpha | Q16665 | 38 | 440375 | 11589 |
| Cytochrome P450 1B1 | Q16678 | 13 | 0 | 0 |
| Ceramide glucosyltransferase | Q16739 | 43 | 9 | 0 |
| Glutaminyl-peptide cyclotransferase | Q16769 | 102 | 2 | 0 |
| Carbonic anhydrase 9 | Q16790 | 1163 | 216704 | 186 |
| Discoidin domain-containing receptor 2 | Q16832 | 30 | 80 | 3 |
| Membrane primary amine oxidase | Q16853 | 84 | 0 | 0 |
| AP2-associated protein kinase 1 | Q2M2I8 | 14 | 14 | 1 |
| Tubulin beta-8 chain | Q3ZCM7 | 25 | 5 | 0 |
| Mitogen-activated protein kinase kinase kinase 19 | Q56UN5 | 23 | 0 | 0 |
| Free fatty acid receptor 4 | Q5NUL3 | 20 | 3 | 0 |
| Leucine-rich repeat serine/threonine-protein kinase 2 | Q5S007 | 264 | 0 | 0 |
| Serine/threonine-protein kinase MRCK alpha | Q5VT25 | 84 | 17 | 0 |
| Long-chain fatty acid transport protein 4 | Q6P1M0 | 11 | 5 | 0 |
| Long-chain fatty acid transport protein 1 | Q6PCB7 | 34 | 0 | 0 |
| Tubulin alpha-3E chain | Q6PEY2 | 25 | 5 | 0 |
| Neutral cholesterol ester hydrolase 1 | Q6PIU2 | 14 | 1 | 0 |
| Dipeptidyl peptidase 8 | Q6V1X1 | 201 | 2 | 0 |
| Neuropeptide S receptor | Q6W5P4 | 44 | 204064 | 4638 |
| 5-hydroxytryptamine receptor 3D | Q70Z44 | 148 | 15 | 0 |
| Tubulin alpha-1A chain | Q71U36 | 25 | 373273 | 14931 |
| Serine/threonine-protein kinase MARK2 | Q7KZI7 | 106 | 306221 | 2889 |
| Serine/threonine-protein kinase TAO1 | Q7L7X3 | 125 | 1 | 0 |
| Ribonucleoside-diphosphate reductase subunit M2 B | Q7LG56 | 35 | 365055 | 10430 |
| Transient receptor potential cation channel subfamily M member 8 | Q7Z2W7 | 40 | 4 | 0 |
| Probable G-protein coupled receptor 142 | Q7Z601 | 73 | 0 | 0 |
| Dipeptidyl peptidase 9 | Q86TI2 | 151 | 2 | 0 |
| Serine/threonine-protein kinase pim-3 | Q86V86 | 240 | 0 | 0 |
| Histone-arginine methyltransferase CARM1 | Q86X55 | 27 | 372386 | 13792 |
| Homeodomain-interacting protein kinase 1 | Q86Z02 | 10 | 375576 | 37558 |
| Calcium/calmodulin-dependent protein kinase type 1D | Q8IU85 | 39 | 17 | 0 |
| Ras guanyl-releasing protein 3 | Q8IV61 | 34 | 11 | 0 |
| Mitogen-activated protein kinase kinase kinase kinase 3 | Q8IVH8 | 13 | 0 | 0 |
| MAP kinase-activated protein kinase 5 | Q8IW41 | 48 | 371328 | 7736 |
| NAD-dependent protein deacetylase sirtuin-2 | Q8IXJ6 | 34 | 363995 | 10706 |
| Carbonic anhydrase 13 | Q8N1Q1 | 112 | 0 | 0 |
| Secreted frizzled-related protein 1 | Q8N474 | 64 | 0 | 0 |
| Misshapen-like kinase 1 | Q8N4C8 | 171 | 217426 | 1271 |
| Calcium/calmodulin-dependent protein kinase kinase 1 | Q8N5S9 | 12 | 437689 | 36474 |
| Kynurenine/alpha-aminoadipate aminotransferase, mitochondrial | Q8N5Z0 | 27 | 0 | 0 |
| Homeodomain-interacting protein kinase 4 | Q8NE63 | 158 | 0 | 0 |
| Phosphatidylinositol 3-kinase catalytic subunit type 3 | Q8NEB9 | 10 | 156875 | 15688 |
| Transient receptor potential cation channel subfamily V member 1 | Q8NER1 | 1103 | 313534 | 284 |
| Tubulin--tyrosine ligase | Q8NG68 | 10 | 0 | 0 |
| Phosphatidylinositol 5-phosphate 4-kinase type-2 gamma | Q8TBX8 | 10 | 0 | 0 |
| Phosphoethanolamine/phosphocholine phosphatase | Q8TCT1 | 25 | 282368 | 11295 |
| Prokineticin receptor 1 | Q8TCW9 | 13 | 0 | 0 |
| Mitogen-activated protein kinase 15 | Q8TD08 | 14 | 15176 | 1084 |
| Serine/threonine-protein kinase BRSK1 | Q8TDC3 | 61 | 260789 | 4275 |
| Mucolipin-3 | Q8TDD5 | 10 | 215587 | 21559 |
| Hydroxycarboxylic acid receptor 2 | Q8TDS4 | 298 | 54 | 0 |
| G-protein coupled bile acid receptor 1 | Q8TDU6 | 186 | 1 | 0 |
| Glucose-dependent insulinotropic receptor | Q8TDV5 | 403 | 3 | 0 |
| Histone-lysine N-methyltransferase, H3 lysine-79 specific | Q8TEK3 | 22 | 1 | 0 |
| Serine/threonine-protein kinase haspin | Q8TF76 | 18 | 2 | 0 |
| G protein-coupled receptor kinase 7 | Q8WTQ7 | 10 | 0 | 0 |
| Scavenger receptor class B member 1 | Q8WTV0 | 37 | 1 | 0 |
| Histone deacetylase 7 | Q8WUI4 | 252 | 409497 | 1625 |
| Gamma-secretase subunit APH-1B | Q8WW43 | 181 | 2 | 0 |
| G2/mitotic-specific cyclin-B3 | Q8WWL7 | 237 | 282 | 1 |
| 5-hydroxytryptamine receptor 3C | Q8WXA8 | 148 | 15 | 0 |
| Nicastrin | Q92542 | 181 | 357372 | 1974 |
| Dual specificity tyrosine-phosphorylation-regulated kinase 2 | Q92630 | 38 | 22 | 1 |
| Lysophosphatidic acid receptor 1 | Q92633 | 79 | 16 | 0 |
| Estrogen receptor beta | Q92731 | 1238 | 421349 | 340 |
| Histone deacetylase 2 | Q92769 | 538 | 436922 | 812 |
| Growth hormone secretagogue receptor type 1 | Q92847 | 942 | 25 | 0 |
| Mitogen-activated protein kinase kinase kinase kinase 1 | Q92918 | 11 | 358028 | 32548 |
| Bcl2 antagonist of cell death | Q92934 | 23 | 501128 | 21788 |
| KiSS-1 receptor | Q969F8 | 79 | 0 | 0 |
| Histone deacetylase 10 | Q969S8 | 299 | 309741 | 1036 |
| Melanin-concentrating hormone receptor 2 | Q969V1 | 61 | 27 | 0 |
| Gamma-secretase subunit APH-1A | Q96BI3 | 181 | 2 | 0 |
| Histone deacetylase 11 | Q96DB2 | 255 | 64 | 0 |
| NAD-dependent protein deacetylase sirtuin-1 | Q96EB6 | 64 | 373540 | 5837 |
| Aurora kinase B | Q96GD4 | 1053 | 334406 | 318 |
| Carboxypeptidase B2 | Q96IY4 | 54 | 215009 | 3982 |
| Lethal(3)malignant brain tumor-like protein 3 | Q96JM7 | 43 | 0 | 0 |
| Lymphokine-activated killer T-cell-originated protein kinase | Q96KB5 | 27 | 365117 | 13523 |
| Histone-lysine N-methyltransferase EHMT2 | Q96KQ7 | 35 | 263165 | 7519 |
| Egl nine homolog 2 | Q96KS0 | 57 | 373610 | 6555 |
| MAP/microtubule affinity-regulating kinase 4 | Q96L34 | 14 | 272611 | 19472 |
| Mas-related G-protein coupled receptor member X1 | Q96LB2 | 34 | 302933 | 8910 |
| Sentrin-specific protease 8 | Q96LD8 | 25 | 361315 | 14453 |
| Pyroglutamylated RFamide peptide receptor | Q96P65 | 14 | 5 | 0 |
| Testis-specific serine/threonine-protein kinase 2 | Q96PF2 | 30 | 0 | 0 |
| NADPH oxidase 5 | Q96PH1 | 15 | 0 | 0 |
| Serine/threonine-protein kinase SMG1 | Q96Q15 | 19 | 14 | 1 |
| PAS domain-containing serine/threonine-protein kinase | Q96RG2 | 16 | 0 | 0 |
| Bile acid receptor | Q96RI1 | 289 | 372669 | 1290 |
| Trace amine-associated receptor 1 | Q96RJ0 | 170 | 355123 | 2089 |
| Calcium/calmodulin-dependent protein kinase kinase 2 | Q96RR4 | 20 | 25 | 1 |
| Solute carrier family 22 member 12 | Q96S37 | 10 | 0 | 0 |
| SRSF protein kinase 1 | Q96SB4 | 49 | 157032 | 3205 |
| Sodium channel protein type 2 subunit alpha | Q99250 | 19 | 5 | 0 |
| Sphingosine 1-phosphate receptor 3 | Q99500 | 227 | 229584 | 1011 |
| Sortilin | Q99523 | 11 | 13 | 1 |
| Mitogen-activated protein kinase kinase kinase 14 | Q99558 | 16 | 320493 | 20031 |
| P2X purinoceptor 7 | Q99572 | 1351 | 90644 | 67 |
| Mitogen-activated protein kinase kinase kinase 5 | Q99683 | 38 | 404556 | 10646 |
| Monoglyceride lipase | Q99685 | 122 | 25 | 0 |
| Melanin-concentrating hormone receptor 1 | Q99705 | 2217 | 1087 | 0 |
| Sigma non-opioid intracellular receptor 1 | Q99720 | 1338 | 6 | 0 |
| Equilibrative nucleoside transporter 1 | Q99808 | 164 | 0 | 0 |
| Endothelial PAS domain-containing protein 1 | Q99814 | 11 | 419171 | 38106 |
| Smoothened homolog | Q99835 | 262 | 1 | 0 |
| Chymotrypsin-C | Q99895 | 32 | 16 | 1 |
| Tubulin alpha-1C chain | Q9BQE3 | 25 | 19 | 1 |
| Sentrin-specific protease 7 | Q9BQF6 | 24 | 323426 | 13476 |
| Eukaryotic translation initiation factor 2-alpha kinase 1 | Q9BQI3 | 26 | 289471 | 11134 |
| Serine/threonine-protein kinase RIO1 | Q9BRS2 | 41 | 0 | 0 |
| MAP kinase-interacting serine/threonine-protein kinase 1 | Q9BUB5 | 20 | 228134 | 11407 |
| Tubulin beta-6 chain | Q9BUF5 | 25 | 5 | 0 |
| Tubulin beta-2B chain | Q9BVA1 | 25 | 5 | 0 |
| Serine/threonine-protein kinase RIO2 | Q9BVS4 | 35 | 0 | 0 |
| Cyclin-dependent kinase 19 | Q9BWU1 | 18 | 0 | 0 |
| Succinate receptor 1 | Q9BXA5 | 41 | 10 | 0 |
| Testis-specific serine/threonine-protein kinase 1 | Q9BXA7 | 53 | 23 | 0 |
| Histone deacetylase 8 | Q9BY41 | 468 | 64815 | 138 |
| Angiotensin-converting enzyme 2 | Q9BYF1 | 51 | 2 | 0 |
| Serine/threonine-protein kinase 33 | Q9BYT3 | 30 | 331884 | 11063 |
| Tryptase delta | Q9BZJ3 | 24 | 0 | 0 |
| Serine/threonine-protein kinase D2 | Q9BZL6 | 131 | 35 | 0 |
| Neuropeptide FF receptor 1 | Q9GZQ6 | 25 | 8 | 0 |
| Sentrin-specific protease 6 | Q9GZR1 | 27 | 373429 | 13831 |
| Egl nine homolog 1 | Q9GZT9 | 141 | 373608 | 2650 |
| NUAK family SNF1-like kinase 2 | Q9H093 | 12 | 4 | 0 |
| Serine/threonine-protein kinase SIK2 | Q9H0K1 | 24 | 0 | 0 |
| Sphingosine 1-phosphate receptor 5 | Q9H228 | 179 | 228861 | 1279 |
| P2Y purinoceptor 12 | Q9H244 | 690 | 4 | 0 |
| STE20-like serine/threonine-protein kinase | Q9H2G2 | 163 | 322384 | 1978 |
| Tankyrase-2 | Q9H2K2 | 108 | 16 | 0 |
| Serine/threonine-protein kinase TAO3 | Q9H2K8 | 13 | 0 | 0 |
| Homeodomain-interacting protein kinase 2 | Q9H2X6 | 219 | 422621 | 1930 |
| Histamine H4 receptor | Q9H3N8 | 607 | 230442 | 380 |
| Tyrosine-protein kinase Srms | Q9H3Y6 | 47 | 0 | 0 |
| Homeodomain-interacting protein kinase 3 | Q9H422 | 13 | 376987 | 28999 |
| Serine/threonine-protein kinase PLK3 | Q9H4B4 | 154 | 370892 | 2408 |
| Tubulin beta-1 chain | Q9H4B7 | 43 | 20 | 0 |
| Elongation of very long chain fatty acids protein 6 | Q9H5J4 | 74 | 0 | 0 |
| Egl nine homolog 3 | Q9H6Z9 | 56 | 373610 | 6672 |
| Prostaglandin E synthase 2 | Q9H7Z7 | 19 | 3 | 0 |
| Dual specificity protein kinase CLK4 | Q9HAZ1 | 588 | 1040 | 2 |
| Transient receptor potential cation channel subfamily V member 4 | Q9HBA0 | 12 | 914 | 76 |
| Peptide deformylase, mitochondrial | Q9HBH1 | 26 | 0 | 0 |
| MAP kinase-interacting serine/threonine-protein kinase 2 | Q9HBH9 | 164 | 209191 | 1276 |
| Lysophosphatidic acid receptor 2 | Q9HBW0 | 46 | 16 | 0 |
| Serine/threonine-protein kinase Sgk2 | Q9HBY8 | 95 | 61 | 1 |
| DNA dC->dU-editing enzyme APOBEC-3G | Q9HC16 | 15 | 397402 | 26493 |
| Nucleotide-binding oligomerization domain-containing protein 2 | Q9HC29 | 22 | 362162 | 16462 |
| G-protein coupled receptor 35 | Q9HC97 | 89 | 288900 | 3246 |
| Non-lysosomal glucosylceramidase | Q9HCG7 | 12 | 0 | 0 |
| Casein kinase I isoform gamma-1 | Q9HCP0 | 90 | 18 | 0 |
| Dual 3',5'-cyclic-AMP and -GMP phosphodiesterase 11A | Q9HCR9 | 38 | 0 | 0 |
| Leukotriene B4 receptor 2 | Q9NPC1 | 67 | 0 | 0 |
| NADPH oxidase 4 | Q9NPH5 | 75 | 209190 | 2789 |
| Dual specificity tyrosine-phosphorylation-regulated kinase 4 | Q9NR20 | 33 | 0 | 0 |
| Sphingosine kinase 2 | Q9NRA0 | 20 | 32 | 2 |
| Tryptase gamma | Q9NRR2 | 26 | 0 | 0 |
| BMP-2-inducible protein kinase | Q9NSY1 | 11 | 13 | 1 |
| NAD-dependent protein deacetylase sirtuin-3, mitochondrial | Q9NTG7 | 24 | 20 | 1 |
| Interleukin-1 receptor-associated kinase 4 | Q9NWZ3 | 173 | 0 | 0 |
| Voltage-dependent calcium channel subunit alpha-2/delta-2 | Q9NY47 | 18 | 0 | 0 |
| Low affinity sodium-glucose cotransporter | Q9NY91 | 25 | 0 | 0 |
| Sphingosine kinase 1 | Q9NYA1 | 74 | 63952 | 864 |
| Toll-like receptor 7 | Q9NYK1 | 112 | 51 | 0 |
| Mitogen-activated protein kinase kinase kinase MLT | Q9NYL2 | 53 | 14 | 0 |
| Serine/threonine-protein kinase PLK2 | Q9NYY3 | 53 | 14 | 0 |
| Gamma-secretase subunit PEN-2 | Q9NZ42 | 183 | 2 | 0 |
| Eukaryotic translation initiation factor 2-alpha kinase 3 | Q9NZJ5 | 55 | 418430 | 7608 |
| Group IIE secretory phospholipase A2 | Q9NZK7 | 12 | 0 | 0 |
| Voltage-dependent T-type calcium channel subunit alpha-1I | Q9P0X4 | 23 | 2 | 0 |
| Serine/threonine-protein kinase pim-2 | Q9P1W9 | 391 | 30 | 0 |
| DNA (cytosine-5)-methyltransferase 3B | Q9UBC3 | 10 | 420756 | 42076 |
| SUMO-activating enzyme subunit 1 | Q9UBE0 | 17 | 287372 | 16904 |
| Serine/threonine-protein kinase NLK | Q9UBE8 | 26 | 201567 | 7753 |
| Phosphatidylinositol 4-kinase beta | Q9UBF8 | 29 | 156867 | 5409 |
| P2X purinoceptor 2 | Q9UBL9 | 37 | 18 | 0 |
| Histone deacetylase 6 | Q9UBN7 | 770 | 217797 | 283 |
| Gamma-aminobutyric acid type B receptor subunit 1 | Q9UBS5 | 18 | 213667 | 11870 |
| SUMO-activating enzyme subunit 2 | Q9UBT2 | 17 | 287372 | 16904 |
| Appetite-regulating hormone | Q9UBU3 | 38 | 33 | 1 |
| Protein DBF4 homolog A | Q9UBU7 | 22 | 280341 | 12743 |
| Cathepsin F | Q9UBX1 | 22 | 9 | 0 |
| Lysophosphatidic acid receptor 3 | Q9UBY5 | 51 | 6 | 0 |
| Serine/threonine-protein kinase 17A | Q9UEE5 | 195 | 13 | 0 |
| Ephrin type-A receptor 6 | Q9UF33 | 10 | 11 | 1 |
| Small conductance calcium-activated potassium channel protein 3 | Q9UGI6 | 13 | 0 | 0 |
| Poly [ADP-ribose] polymerase 2 | Q9UGN5 | 35 | 93 | 3 |
| Acid-sensing ion channel 3 | Q9UHC3 | 12 | 332230 | 27686 |
| Serine/threonine-protein kinase TBK1 | Q9UHD2 | 127 | 319717 | 2517 |
| Dipeptidyl peptidase 2 | Q9UHL4 | 181 | 1 | 0 |
| Leucyl-cystinyl aminopeptidase | Q9UIQ6 | 20 | 17 | 1 |
| Ribosomal protein S6 kinase alpha-6 | Q9UK32 | 10 | 141889 | 14189 |
| TRAF2 and NCK-interacting protein kinase | Q9UKE5 | 29 | 11 | 0 |
| Urotensin-2 receptor | Q9UKP6 | 156 | 10 | 0 |
| Histone deacetylase 9 | Q9UKV0 | 253 | 361115 | 1427 |
| Carbonic anhydrase 14 | Q9ULX7 | 254 | 0 | 0 |
| ALK tyrosine kinase receptor | Q9UM73 | 435 | 362511 | 833 |
| A disintegrin and metalloproteinase with thrombospondin motifs 5 | Q9UNA0 | 179 | 64735 | 362 |
| Chymotrypsin-like elastase family member 1 | Q9UNI1 | 13 | 1 | 0 |
| ATP-binding cassette sub-family G member 2 | Q9UNQ0 | 69 | 192799 | 2794 |
| Aurora kinase C | Q9UQB9 | 48 | 15 | 0 |
| Histone deacetylase 5 | Q9UQL6 | 265 | 321991 | 1215 |
| Calcium/calmodulin-dependent protein kinase type II subunit alpha | Q9UQM7 | 31 | 386818 | 12478 |
| Short transient receptor potential channel 6 | Q9Y210 | 15 | 90 | 6 |
| cAMP and cAMP-inhibited cGMP 3',5'-cyclic phosphodiesterase 10A | Q9Y233 | 623 | 322363 | 517 |
| Nucleotide-binding oligomerization domain-containing protein 1 | Q9Y239 | 60 | 370544 | 6176 |
| RAC-gamma serine/threonine-protein kinase | Q9Y243 | 162 | 72 | 0 |
| Heparanase | Q9Y251 | 72 | 3 | 0 |
| Cysteinyl leukotriene receptor 1 | Q9Y271 | 100 | 4 | 0 |
| Carbonic anhydrase 5B, mitochondrial | Q9Y2D0 | 63 | 0 | 0 |
| Nischarin | Q9Y2I1 | 29 | 0 | 0 |
| Tyrosine-protein phosphatase non-receptor type 22 | Q9Y2R2 | 39 | 291630 | 7478 |
| G-protein coupled receptor 55 | Q9Y2T6 | 88 | 289427 | 3289 |
| Mitogen-activated protein kinase kinase kinase 2 | Q9Y2U5 | 12 | 333904 | 27825 |
| Sodium- and chloride-dependent glycine transporter 2 | Q9Y345 | 36 | 6 | 0 |
| N-acetylated-alpha-linked acidic dipeptidase 2 | Q9Y3Q0 | 14 | 0 | 0 |
| Dual specificity tyrosine-phosphorylation-regulated kinase 1B | Q9Y463 | 174 | 175733 | 1010 |
| Sn1-specific diacylglycerol lipase alpha | Q9Y4D2 | 16 | 7 | 0 |
| Mitogen-activated protein kinase kinase kinase kinase 5 | Q9Y4K4 | 237 | 352747 | 1488 |
| Histamine H3 receptor | Q9Y5N1 | 2409 | 23 | 0 |
| NADPH oxidase 1 | Q9Y5S8 | 57 | 16097 | 282 |
| Photoreceptor-specific nuclear receptor | Q9Y5X4 | 23 | 359596 | 15635 |
| Neuropeptide FF receptor 2 | Q9Y5X5 | 25 | 2 | 0 |
| Endothelial lipase | Q9Y5X9 | 11 | 0 | 0 |
| Prostaglandin D2 receptor 2 | Q9Y5Y4 | 1039 | 2 | 0 |
| Suppressor of tumorigenicity 14 protein | Q9Y5Y6 | 106 | 901 | 9 |
| Sodium channel protein type 10 subunit alpha | Q9Y5Y9 | 69 | 90637 | 1314 |
| Beta-secretase 2 | Q9Y5Z0 | 260 | 192894 | 742 |
| Nuclear receptor corepressor 2 | Q9Y618 | 50 | 486556 | 9731 |
| NF-kappa-B essential modulator | Q9Y6K9 | 13 | 441285 | 33945 |
| Casein kinase I isoform gamma-3 | Q9Y6M4 | 63 | 0 | 0 |
| Nuclear receptor coactivator 3 | Q9Y6Q9 | 10 | 471857 | 47186 |

**Table S3**. The number of actives and experimental inactives for the classes contained in the models.


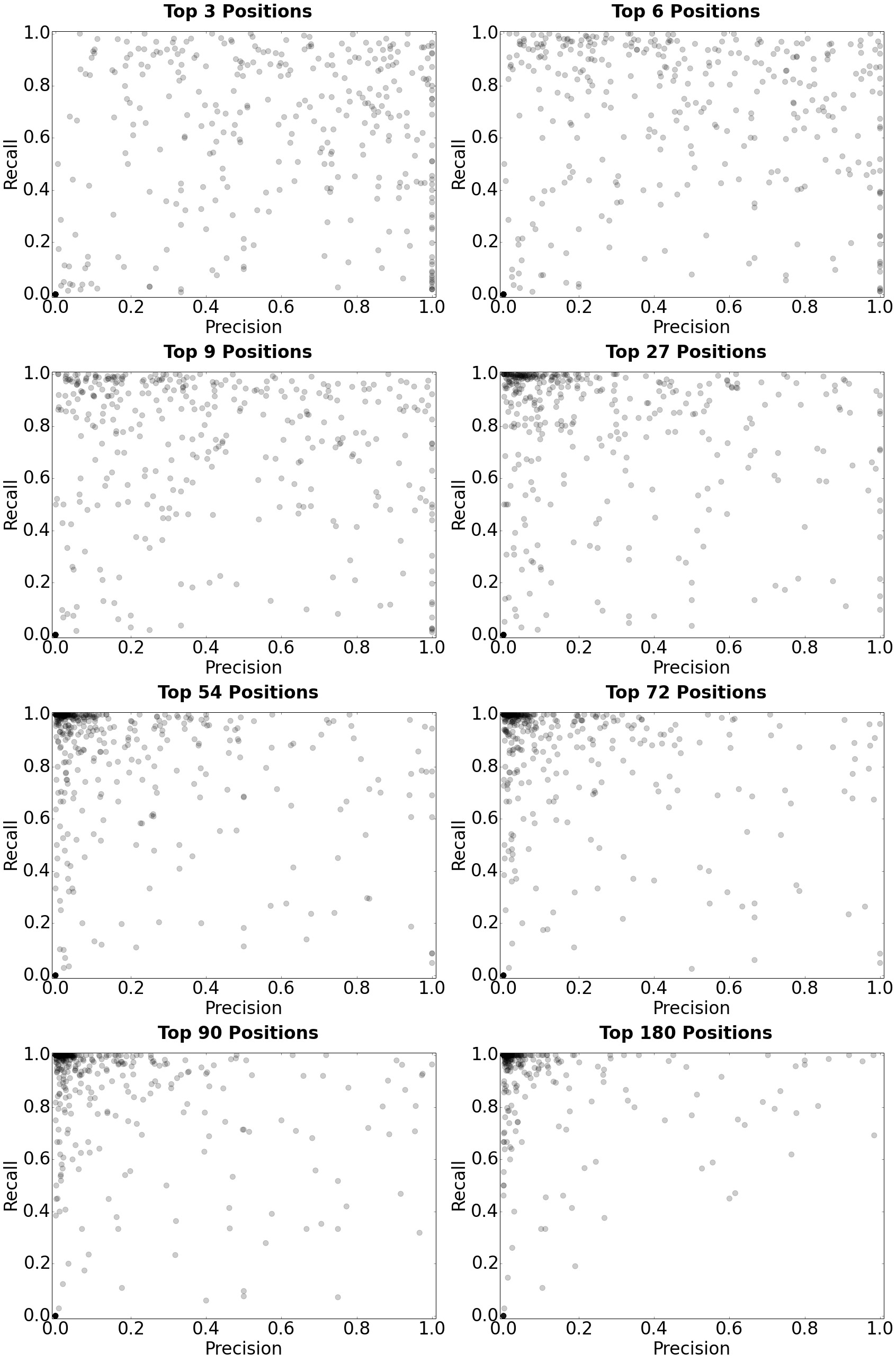


**Figure S1.** Precision and recall distributions achieved by the activity-only model based on different top-*k* positions to define activity. The plots show that increasing the top-*k* positions increases the recall at a cost of decreasing precision
